# Gain-of-Sensitivity Mutations in a Trim5-Resistant Primary Isolate of Pathogenic SIV Identify Two Independent Conserved Determinants of Trim5α Specificity

**DOI:** 10.1371/journal.ppat.1003352

**Published:** 2013-05-09

**Authors:** Kevin R. McCarthy, Aaron G. Schmidt, Andrea Kirmaier, Allison L. Wyand, Ruchi M. Newman, Welkin E. Johnson

**Affiliations:** 1 Harvard Program in Virology, Harvard Medical School, Boston, Massachusetts, United States of America; 2 Biology Department, Boston College, Chestnut Hill, Massachusetts, United States of America; 3 Laboratory of Molecular Medicine, Children's Hospital, Harvard Medical School, Boston, Massachusetts, United States of America; 4 Broad Institute of MIT and Harvard, Cambridge, Massachusetts, United States of America; Fred Hutchinson Cancer Research Center, United States of America

## Abstract

Retroviral capsid recognition by Trim5 blocks productive infection. Rhesus macaques harbor three functionally distinct *Trim5* alleles: *Trim5α^Q^*, *Trim5α^TFP^* and *Trim5^CypA^*. Despite the high degree of amino acid identity between *Trim5α^Q^* and *Trim5α^TFP^* alleles, the Q/TFP polymorphism results in the differential restriction of some primate lentiviruses, suggesting these alleles differ in how they engage these capsids. Simian immunodeficiency virus of rhesus macaques (SIVmac) evolved to resist all three alleles. Thus, SIVmac provides a unique opportunity to study a virus in the context of the Trim5 repertoire that drove its evolution in vivo. We exploited the evolved rhesus Trim5α resistance of this capsid to identify gain-of-sensitivity mutations that distinguish targets between the *Trim5α^Q^* and *Trim5α^TFP^* alleles. While both alleles recognize the capsid surface, *Trim5α^Q^* and *Trim5α^TFP^* alleles differed in their ability to restrict a panel of capsid chimeras and single amino acid substitutions. When mapped onto the structure of the SIVmac239 capsid N-terminal domain, single amino acid substitutions affecting both alleles mapped to the *β*-hairpin. Given that none of the substitutions affected Trim5α^Q^ alone, and the fact that the *β*-hairpin is conserved among retroviral capsids, we propose that the *β*-hairpin is a molecular pattern widely exploited by Trim5α proteins. Mutations specifically affecting rhesus Trim5α^TFP^ (without affecting *Trim5α^Q^*) surround a site of conservation unique to primate lentiviruses, overlapping the CPSF6 binding site. We believe targeting this site is an evolutionary innovation driven specifically by the emergence of primate lentiviruses in Africa during the last 12 million years. This modularity in targeting may be a general feature of Trim5 evolution, permitting different regions of the PRYSPRY domain to evolve independent interactions with capsid.

## Author Summary

TRIM5α is an intrinsic immunity protein that blocks retrovirus infection through a specific interaction with the viral capsid. Uniquely among primates, rhesus macaques harbor three functionally distinct kinds of Trim5 alleles: rhTrim5α^TFP^, rhTrim5α^Q^ and rhTrim5^CypA^. SIVmac239, a simian immunodeficiency virus that causes AIDS in rhesus macaques, is resistant to all three, whereas its relative, the human AIDS virus HIV-1, is inhibited by rhTrim5α^TFP^ and rhTrim5α^Q^ alleles. We exploited this difference between these two retroviruses to figure out how Trim5α proteins recognize viral capsids. By combining mutagenesis, structural biology and evolutionary data we determined that both rhTrim5α^TFP^ and rhTrim5α^Q^ recognize a conserved structure common to all retroviral capsids. However, we also found evidence suggesting that rhTrim5α^TFP^ evolved to recognize an additional target that is specifically conserved among primate immunodeficiency viruses. Molecular evolutionary analysis indicates that this expanded function appeared in a common ancestor of modern African monkeys sometime between 9–12 million years ago, and that it thereafter continued to be modified by strong evolutionary pressure. Our results provide insight into the evolutionary flexibility of Trim5α-capsid interactions, and support the notion that viruses related to modern HIV and SIV have been present in Africa for millions of years.

## Introduction

The anti-retroviral activity of Trim5α was discovered in a screen to identify rhesus macaque cDNAs conferring resistance to HIV-1 replication [1]. Antiretroviral activity has since been demonstrated for a large number of primate Trim5 orthologs, including prosimians, as well as homologs from cow and rabbit [2], [3], [4], [5]. While no single ortholog of Trim5 universally restricts all retroviruses, the collective breadth of restriction, coupled with the observation that some orthologs can restrict viruses from two or more genera, suggests that Trim5 recognizes a conserved, pathogen-associated molecular pattern common to members of the *Retroviridae* [2], [6], [7].

Trim5α is composed of four domains: the RING, the B-Box and the Coiled-coil domains, which make up the tripartite RBCC of TRIM proteins, and a C-terminal PRYSPRY domain [8], [9]. The PRYSPRY domain is thought to recognize the viral capsid [1], [10], [11]. In the case of lentiviruses, the cone-shaped capsid is composed of 12 pentamers and approximately 200 hexamers, each in turn comprised of identical copies of monomeric capsid (CA) protein [12], [13]. An HIV-1 CA monomer has two *α*-helical domains connected by a flexible linker [14]. The N-terminal domain makes up the outer surface of the capsid and mediates interactions with cellular cofactors [15], [16], [17], [18], [19], [20], [21].

Comparisons between reported CA structures from viruses representing five *Orthoretrovirinae* genera show that the overall architecture of the N-terminal domain is conserved, despite little conservation of protein sequence. All reported retroviral N-terminal domain structures contain a conserved five *α*-helix core, from which a conserved surface feature, the *β*-hairpin, protrudes into the cytoplasm. Structural variation can be found among additional features on the CA surface. These differences include the presence and arrangement of 1–2 additional α-helices and/or the presence of an extended loop connecting helices 4 and 5 (4–5 loop) [22], [23], [24], [25], [26], [27].

Reports suggest that multiple sites within retroviral CAs modulate Trim5α sensitivity [28], [29], [30], [31], [32], [33], [34], [35], [36], [37], [38], [39], [40], [41], [42], [43], [44], [45], [46], [47], [48], [49], [50], [51], [52]. The majority of these sites map to the N-terminal domain and are enriched within the CA surface features. Perplexingly, engineered CA mutations, naturally occurring variants, and escape mutations can have similar phenotypes even when separated by distances in excess of 25 Å. Understanding how these sites relate to one another is critically important for defining how Trim5α recognizes retroviral capsids, and how viruses evolve to evade Trim5α restriction.

We previously reported that the *Trim5* locus of rhesus macaques (*Macaca mulatta*) is highly polymorphic, and that the different allelic lineages of rhesus *Trim5* (*rhTrim5*) have been maintained by long-term balancing selection [53], [54]. Based on functional assays and gene association studies, rhTrim5 alleles can be grouped into 3 classes, rhTrim5α^TFP^, rhTrim5α^Q^ and rhTrim5^CypA^ [31], [55], [56], [57], [58], [59], [60]. When tested against a panel of primate lentiviruses, the 3 alleles give differing patterns of restriction [31], [53], [57] – an indication that rhTrim5 has at least 3 distinct (or incompletely overlapping) targets on the lentiviral CA protein.

SIVmac emerged in captive macaque colonies in the 1970s, most likely the result of an unintentional interspecies transmission of SIV from sooty mangabeys (SIVsm) [61], [62], [63], [64]. We previously reported that SIVsm isolates are resistant to rhTrim5α^Q^, but sensitive to rhTrim5α^TFP^ and rhTrim5^CypA^ alleles [31]. Because rhTrim5α^TFP^, rhTrim5α^Q^ and rhTrim5^CypA^ likely have deferring targets within CA and because all are present at moderate-to-high frequency, emergence of SIVmac in rhesus macaque colonies required adaptations permitting simultaneous resistance to all three. Thus, comparisons between SIVmac and other restricted isolates provide a unique opportunity to understand the basis of recognition by Trim5α proteins and to identify specific features of CA that determine sensitivity and resistance to rhTrim5α-mediated restriction.

The structural basis for CA recognition by rhTrim5^Cyp^ is clear: the cyclophilin A domain (CypA) specifically binds the 4–5 loop [65], [66]. In contrast, rhTrim5α^TFP^ and rhTrim5α^Q^ interact with capsids via a C-terminal PRYSPRY domain, but the basis for capsid recognition by Trim5 PRYSPRY domains remains poorly understood. There are several factors that complicate studies of the interaction. For example, Trim5α destabilizes capsid complexes [10], [11], [67], [68], [69], the nature of the interaction is believed to be high avidity and low affinity [69], [70], [71], [72], the interaction site may extend beyond a single CA monomer or hexamer [11], [67], [70], [72], [73], retroviral capsids and presumably the Trim5α lattice surrounding them have variable morphology and composition [70], [74], and there is considerable diversity among Trim5α orthologs and retroviral CA sequences.

To investigate how Trim5α recognizes retroviral CAs, we combined genetic, phylogenic and structural investigations with an alternative mutational strategy to separate and map the determinants for the differential restriction of HIV-1 and SIVmac by rhTrim5α alleles. The resolution of our mapping, together with the structural determination of the SIVmac239 CA N-terminal domain and consideration of primate lentivirus diversity, allowed us to identify two conserved CA surface elements that appear to be targets of rhTrim5α recognition. The first, the *β*-hairpin, is a structural feature that is present in all reported retroviral CA structures. Mutations in the *β*-hairpin affected targeting by both rhTrim5α^Q^ and rhTrim5α^TFP^ alleles. The second element, a patch of highly conserved amino acids among primate lentivirus CAs, maybe a unique target of the more recently evolved rhTrim5α^TFP^ allele. Strikingly, this patch is a surface-exposed extension of the recently indentified CPSF6 binding site [18]. Therefore, similar to the exploitation of the interaction between cyclophilin A and Nup358 by Trim5^CypA^, it appears that rhTrim5α^TFP^ has evolved to target the binding site of a required cellular cofactor. Taken together, the observations made from investigating the differential breadth and specificities of rhTrim5α alleles have revealed a complex evolutionary relationship between retroviruses and Trim5α orthologues.

## Results

### Differential restriction by the rhesus Trim5α^Q^ and Trim5α^TFP^ alleles

**Figure 1:**
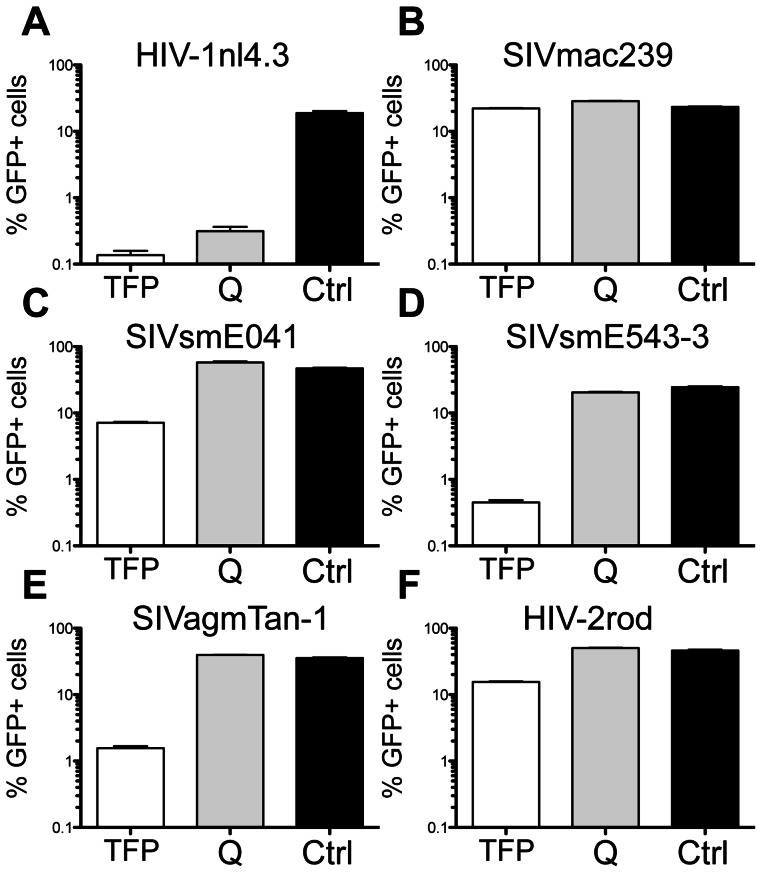
Differential restriction of primate lentiviruses by rhesus Trim5α^TFP^ and Trim5α^Q^ alleles. GFP reporter viruses were used to infect CRFK cells expressing the rhesus Trim5α^TFP^ allele mamu3 (TFP) and the rhesus Trim5α^Q^ allele mamu4 (Q). Infectivity on empty vector control cells is shown (Ctrl). (A) HIV-1nl4.3. (B) SIVmac239. (C) SIVsmE041. (D) SIVsmE543-3. (E) SIVagmTan-1. (F) HIV-2Rod. Infections were done in triplicate. Error bars indicate SEM. These results are representative of at least 3 independent experiments.

Differential restriction by rhTrim5α^Q^ and rhTrim5α^TFP^ has been mapped to a length polymorphism in the PRYSPRY domain (TFP339-341Q) [57]. Despite the fact that the protein sequences are >98% identical, the rhTrim5α^Q^ and rhTrim5α^TFP^ alleles yield different patterns of restriction when tested in parallel against divergent retroviruses [31], [53], [56], [57]. We tested both alleles against multiple primate lentiviruses and found that even among these related viral strains, the rhTrim5α^Q^ and rhTrim5α^TFP^ alleles give different patterns of restriction (Figure 1). Specifically, rhTrim5α^Q^ restricted a human viral isolate, HIV-1nl4.3, but failed to restrict any of the lentiviruses isolated from *Cercopithecine* primates (SIVmac239 from rhesus macaques, SIVsmE041 and SIVsmE543-3 from sooty mangabeys, and SIVagmTAN-1 from African green monkeys) or HIV-2ROD (which originated by cross-species transmission of SIVsm [75]). In contrast, rhTrim5α^TFP^ restricted HIV-1nl4.3, SIVsmE041, SIVsmE543-3, SIVagmTAN-1 and to a lesser extent, HIV-2ROD. Only the rhesus macaque isolate, SIVmac239, was resistant to both alleles. Thus, while both alleles are functional, the differing patterns of restriction are consistent with the hypothesis that rhTrim5α^Q^ and rhTrim5α^TFP^ proteins differ in the way they recognize primate lentivirus CAs.

### Individual surface elements of capsid determine restriction by Trim5α

**Figure 2:**
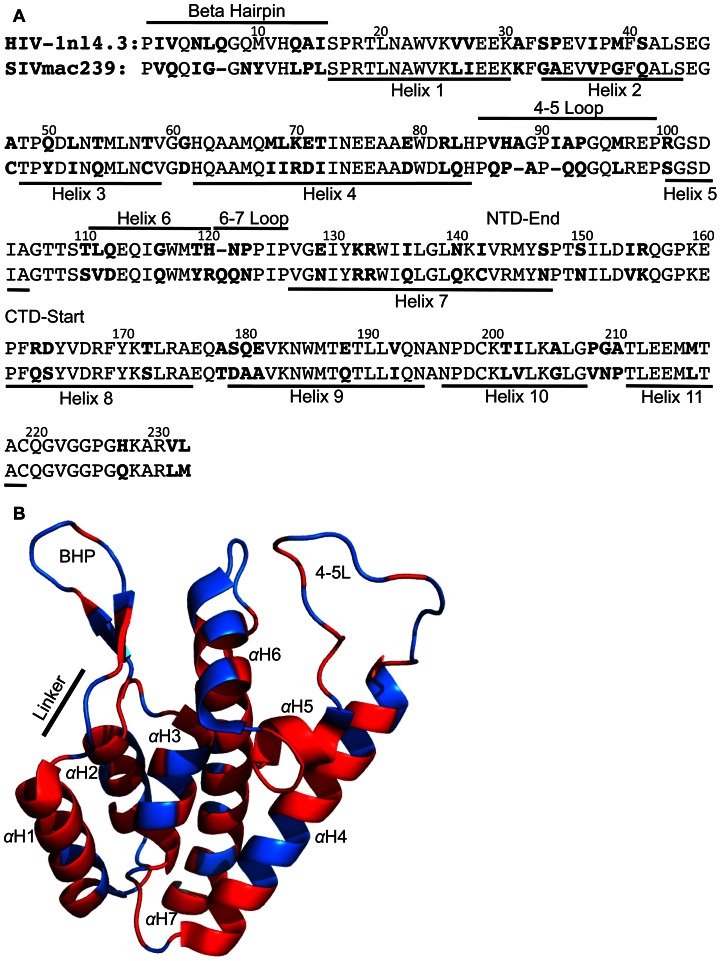
Distribution of amino acid differences between HIV-1nl4.3 and SIVmac239. (A) Sequence alignment of HIV-1nl4.3 and SIVmac239 CAs. Surface features are indicated on the top, internal *α*-helices on the bottom. Amino acid differences between the two viruses are in bold type. (B) Structure of the HIV-1nl4.3 N-terminal domain (PDB: 3GV2). The *β*-hairpin (BHP), the linker connecting the *β*-hairpin to helix 1 (linker), helix 6 (h6) and 4–5 loop (4–5L) are indicated. Additional *α*-helices are numbered αH1-αH7. Residues that are identical in HIV-1nl4.3 and SIvamc239 are in red, residues that differ are in blue.

HIV-1 and SIVmac239 had opposite restriction profiles when tested for restriction on rhTrim5α expressing cells. HIV-1nl4.3 was restricted by both rhTrim5α^TFP^ and rhTrim5α^Q^ alleles, whereas SIVmac239 was resistant to both alleles. At least three lines of evidence support the existence of multiple sites of rhTrim5α recognition within the HIV-1 CA. First, HIV-1 is restricted by both rhTrim5α^TFP^ and rhTrim5α^Q^ alleles while other tested primate lentiviruses are resistant to the rhTrim5α^Q^ allele. Second, attempts to evolve an HIV-1 with resistance to rhTrim5α have not yielded fully resistant viruses [42], while other viruses have successfully evolved resistance to rhTrim5α-mediated restriction with genuine escape mutations both *in vitro* and *in vivo* [30], [31]. Third, mutagenesis approaches in which elements of the SIVmac239 CA were inserted into the HIV-1 CA resulted in rhTrim5α restricted viruses [28], [29], [37], [38]. With 79 amino acid differences between the two viruses (Figure 2A), we hypothesized that isolating each determinant would allow us to resolve the specific amino acids involved in rhTrim5α recognition at each target site. We therefore chose to take an alternative approach, based on identifying gain of sensitivity mutations of the inherently rhTrim5α-resistant SIVmac239 CA. We inserted individual features of the HIV-1nl4.3 CA into the SIVmac239 CA and measured the impact on restriction.

**Figure 3:**
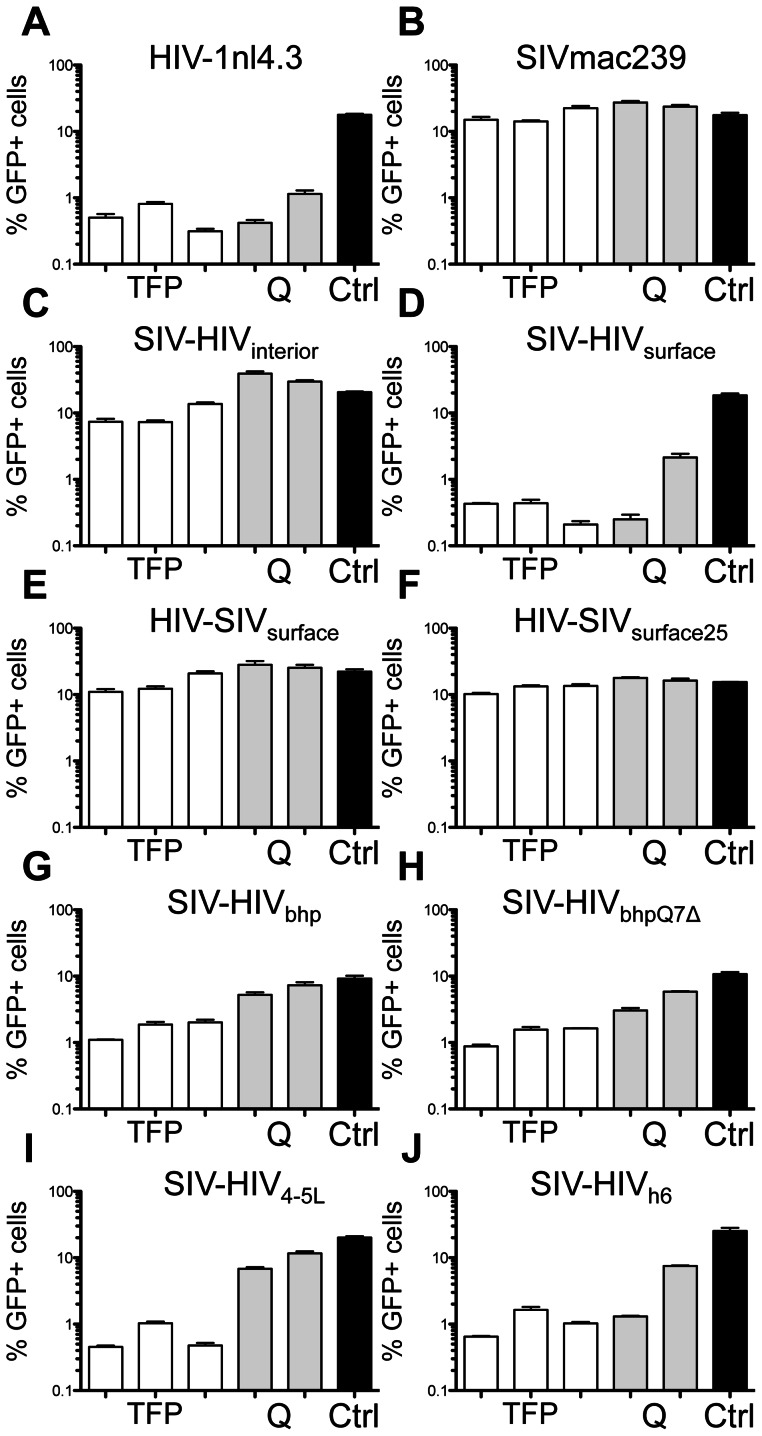
Rhesus Trim5αs recognize the capsid surface. The indicated GFP reporter viruses were used to infect CRFK cells expressing rhesus Trim5α^TFP^ alleles mamu1, mamu2 and mamu3 (TFP) and the Trim5α^Q^ alleles mamu4 and mamu5 (Q). Infectivity on empty vector control cells is shown (Ctrl). (A) HIV-1nl4.3. (B) SIVmac239. (C) SIV-HIV_interior_. (D) SIV-HIV_surface_. (E) HIV-SIV_surface_. (F) HIV-SIV_surface25_. (G) SIV-HIV_bhp_. (H) SIV-HIV_bhpQΔ7_.(I) SIV-HIV_4–5L_. (J) SIV-HIV_h6_. Infections were done in triplicate. Error bars indicate SEM. These results are representative of at least 3 independent experiments.

The ability of Trim5α orthologs to restrict highly divergent retroviruses with little to no sequence identity suggests Trim5α may target conserved, structural elements of CA. All reported retroviral N-terminal domain structures have a conserved five *α*-helix core. To determine whether differences within the five *α*-helix core impact rhTrim5α recognition, we generated SIV-HIV_interior_, by replacing most of the five *α*-helix core of SIVmac239 with that of HIV-1nl4.3. This virus retained the SIVmac239 residues at the first and last amino acid of each *α*-helix (Figure S1). We then tested this virus for restriction by rhTrim5α^TFP^ and rhTrim5α^Q^ alleles. This mutant was 2.3-fold more sensitive to rhTrim5α^TFP^ than the SIVmac239 parent (Figures 3A–3C and Figure S2). This differed markedly from SIV-HIV_surface_, in which three surface elements, the *β*-hairpin, 4–5 loop and helix 6, were derived from HIV-1nl4.3. This virus was restricted by all rhTrim5α alleles tested, at levels similar to HIV-1nl4.3 (Figures 3A–3D).

Because SIV-HIV_surface_ was phenotypically similar to HIV-1nl4.3, we asked whether a reciprocal chimera was sufficient to render HIV-1nl4.3 restriction resistant. Therefore, we replaced the HIV-1nl4.3 CA surface features with the three SIVmac239 surface features (the *β*-hairpin, 4–5 loop and helix 6) to create HIV-SIV_surface_ (Figure S1). This HIV-1 variant differed from HIV-1nl4.3 by 28 amino acids and was highly resistant to restriction by rhTrim5α^TFP^ and rhTrim5α^Q^ alleles (Figure 3E). Within the linker that connects the *β*-hairpin to helix 1, HIV-1nl4.3 and SIVmac239 differ at three positions (amino acids 13–15) (Figure 2 and Figure S1). Using a second HIV-1-SIV chimera, HIV-SIV_surface25_, we determined that these three differences do not influence restriction (Figure 3F). To our knowledge, HIV-SIV_surface_ and HIV-SIV_surface25_ represent the first description of an HIV-1 strain resistant to all allelic classes of rhTrim5. Titration of these viruses and abrogation assays confirm that resistance was not due to saturation of rhTrim5α in the target cell lines (Figures S2 and S3).

To examine the individual contributions of each of the three surface features to restriction, we produced a series of SIVmac239 CAs each grafted with a single HIV-1nl4.3 surface feature. To take into account the fact that the *β*-hairpin is one amino acid shorter in SIVmac239, we generated two SIV variants: SIV-HIV_bhp_, with a full length HIV-1nl4.3 *β*-hairpin, and SIV-HIV_bhpQ7Δ_, with a single amino acid deletion in the HIV-1nl4.3 *β*-hairpin. We also generated SIVmac239 variants with the HIV-1nl4.3 4–5 loop or helix 6 (SIV-HIV_4–5L_ and SIV-HIV_h6_, respectively). Rhesus Trim5α^TFP^ alleles restricted all four of these viruses (SIV-HIV_bhp_, SIV-HIV_bhpQ7Δ_, SIV-HIV_4–5L_, and SIV-HIV_h6_). With the exception of SIV-HIV_h6_, the chimeras had little effect on restriction by rhTrim5α^Q^ (Figure 3G–J). Together, these mutants suggest that the HIV-1 restriction-sensitive and SIVmac239 restriction-resistant phenotypes involve contributions from all three capsid surface features.

### Capsid mutagenesis reveals differences in restriction by Trim5α^TFP^ and Trim5α^Q^

Based on results obtained from the HIV-SIV_surface25_ chimera, we generated a series of SIVmac239 CA mutations in which the amino acid at each of the 25 positions of interest was substituted with the amino acid found at the homologous position in HIV-1nl4.3 (Figures 2A, 3F, S1, S2 and Table 1). Two of the 25 mutations in the SIVmac239 CA, R117H and N123P, resulted in loss of infectivity. Although a His is found at position 117 in HIV-1nl4.3, an Asp is more common among HIV-1 isolates. We found that an SIVmac239 in which R117 was substituted with Asp instead of His retained infectivity (Figure S2).

**Table 1:** Single amino acid mutants reveal differences in restriction by Trim5^TFP^ and Trim5^Q^.

Mutation	SIVmac239 Residue	TFP	Q
V2I	V2	1.21±0.08	1.02±0.14
Q3V	Q3	5.58±0.92	5.42±0.05
I5N	I5	0.70±0.05	0.86±0.08
G6L	G6	8.37±1.46	6.59±2.28
Δ7Q	Δ	7.73±1.35	0.96±0.15
N9Q	N8	1.82±0.21	1.09±0.20
Y10M	Y9	3.41±0.07	0.94±0.11
Q86V	Q85	3.14±0.31	0.99±0.05
P87H	P86	5.73±1.06	0.95±0.17
Δ88A	Δ	1.12±0.10	0.95±0.04
A89G	A87	4.08±0.50	0.94±0.14
Δ91I	Δ	4.53±0.56	0.91±0.22
Q92A	Q89	0.95±0.20	1.00±0.21
Q93P	Q90	2.53±0.28	1.01±0.24
L96M	L93	7.12±0.58	1.03±0.08
S100R	S97	10.80±2.46	0.95±0.20
S110T	S107	1.6±0.23	1.01±0.18
V111L	V108	6.01±0.09	2.11±0.37
D112Q	D109	7.15±0.76	1.39±0.25
Q116G	Q113	2.83±0.16	1.05±0.15
Y119T	Y116	0.73±0.03	0.94±0.07
R120H	R117	N.D.	N.D.
R120N	R117	0.71±0.08	1.04±0.14
Q121Δ	Q118	1.79±0.13	0.91±0.13
Q122N	Q119	0.75±0.07	1.01±0.09
N123P	N120	N.D.	N.D.

The amino acid numbering of mutations corresponds to the alignment in Figure 2A. Numbering corresponding to the SIVmac239 capsid (Accession number M33262) is also provided (column 2). All values are shown as fold-restriction relative to parental SIVmac239. The values are the result of 3 independent experiments, each done in triplicate. The error represents the standard deviation between these 9 infections. N.D - mutant was not infectious and was not analyzed.

The 24 infectious SIVmac239 variants with single amino acid substitutions in CA were tested for sensitivity to restriction by rhTrim5α^TFP^ and rhTrim5α^Q^. Restriction was quantified by determining the level of infectivity relative to SIVmac239 (Table 1). Only two single amino acid substitutions (Q3V and G6L), both in the *β*-hairpin, resulted in gain-of-sensitivity to both rhTrim5α^TFP^ and rhTrim5α^Q^. There were 12 additional mutations that caused gain-of-sensitivity to rhTrim5α^TFP^, but not to rhTrim5α^Q^. These mutations were spread among all three CA surface features. Together these results indicate that the targets of the two alleles partially overlap, and that the overlap involves elements within the *β*-hairpin. The observation that a large number of residues outside of the *β*-hairpin exclusively affect rhTrim5α^TFP^ without altering rhTrim5α^Q^ sensitivity raises the possibility that rhTrim5α^TFP^ either has a larger footprint on the CA surface than rhTrim5α^Q^, or that it has the capacity to target more than one determinant in CA. Most notably, there were no mutations that affected only the rhTrim5α^Q^ allele (that is, none of the mutations tested caused gain-of-sensitivity to rhTrim5α^Q^ but not to rhTrim5α^TFP^). This trend was mirrored among the 14 other viruses tested, including both naturally occurring viruses and chimeric viruses generated for this study (Figures 1 and 3).

### Structure of the SIVmac239 CA N-terminal domain

**Figure 4:**
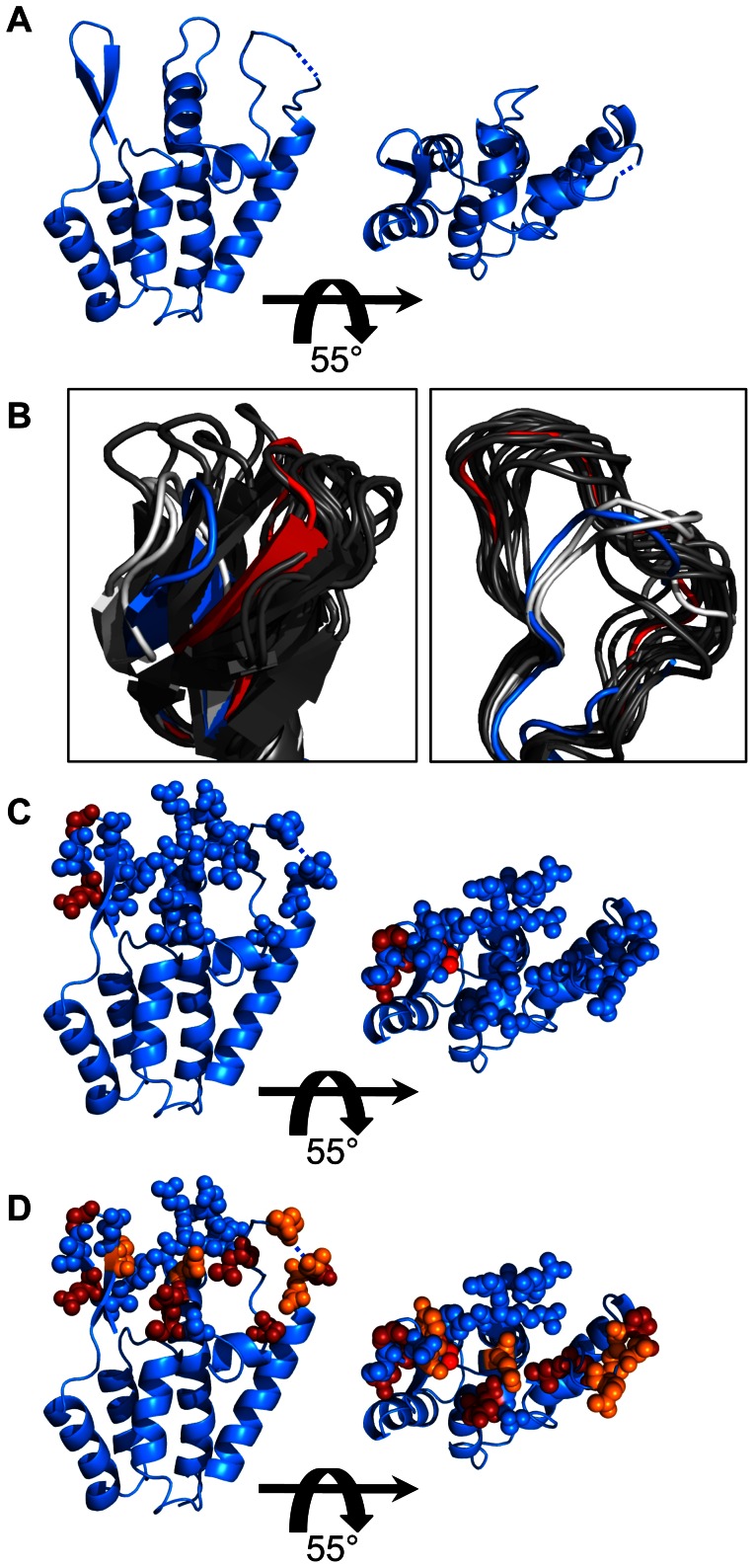
Structure of the SIVmac239 Capsid N-terminal domain. (A) Structure of the SIVmac239 CA N-terminal domain at 2.9 Å resolution. There was no clear density for Pro88, and thus, it was omitted from the structure. A dashed line is used to indicate its place. (B) Comparison of the SIVmac239 *β*-hairpin and 4–5 loop to all other wild type HIV-1 and HIV-2 X-ray structures deposited in the PDB. HIV-1 structures are colored dark gray, except PDB: 2X2D, which is colored red (and used in all subsequent comparisons). HIV-2 structures are colored light gray, and the SIVmac239 N-terminal domain is colored blue. (C and D) Locations of amino acids mutations associated with rhesus Trim5α^Q^ (C) and rhesus Trim5α^TFP^ (D) restriction from Table 1. Blue spheres indicate amino acid differences that do not impact Trim5α restriction. Orange spheres show the location of mutations associated with 2.5–5 fold gains in sensitivity to rhesus Trim5α relative to SIVmac239. Red spheres indicate positions associated with >5 fold gains in sensitivity to rhesus Trim5α. Images created in PyMol.

To provide a relevant structural context for evaluating the mutagenesis results, we determined the structure of the SIVmac239 CA N-terminal domain (Figures 4A, S4, S5 and Table S1). The SIVmac239 CA N-terminal domain was very similar to reported structures of HIV-1 (PDB: 2X2D) (RMSD at Cα positions: 2.29 Å) and HIV-2 (PDB: 2WLV) (RMSD at Cα positions: 1.42 Å) (calculations used SuperPose [76]). In particular, the five *α*-helices of the SIVmac239 N-terminal domain core did not deviate from those of HIV-1 or HIV-2, consistent with the observation that the SIV-HIV_interior_ chimera remained largely resistant to restriction (Figure 3C).

Since the amino acids governing rhTrim5α restriction mapped to the CA surface, we were particularly interested in structural differences between SIVmac239 and HIV-1 in the *β*-hairpin, 4–5 loop and helix 6. We compared the SIVmac239 CA N-terminal domain structure to all of the previously reported wild type HIV-1 and HIV-2 CA N-terminal domain structures in which the surface features were properly folded (Figure 4B and Figure S5). This dataset includes structures of CA monomers, CA monomers from cyclophilin A bound HIV-1 CAs, HIV-1 hexamers and HIV-1 pentamers. From this analysis, we found a clear distinction between the HIV-1 structures and those of the more closely related HIV-2 and SIVmac239. Specifically, the 4–5 loops and *β*-hairpins formed two clusters; one composed of HIV-1 structures, and the other composed of SIVmac239 and HIV-2 structures. Measurements between the HIV-1 Cα of Gly94 or Gln95 and the corresponding Gly91 and Gln92 of SIVmac239 indicate that these two groups are separated by 3.3–11 Å in the structural alignment. Similarly, measurements between the Cα of HIV-1 Gly8 and the homologous SIVmac239/HIV-2 Gly7 show the two groups are separated by 4–8.5 Å in the structural alignment (Figure 4B). These CA structural differences may help to explain the observed changes in restriction between the reciprocal SIV-HIV_surface_ and HIV-SIV_surface_ chimeras (Figures 3D and 3E).

To determine the spatial arrangement of the single amino acid substitutions associated with rhTrim5α restriction, we mapped the restriction data for rhTrim5α^Q^ and rhTrim5α^TFP^ onto the structure of the SIVmac239 N-terminal domain (Figures 4C and 4D respectively) as well as the structure of the HIV-1 CA hexamer (Figure S6). The two individual point mutations associated with rhTrim5α^Q^ restriction were confined to the *β*-hairpin and were within 10 Å of each other. This differed from rhTrim5α^TFP^, which in addition to being affected by the same two sites in the *β*-hairpin, also recognized amino acid substitutions outside the *β*-hairpin, spanning approximately 30 Å of the CA surface.

### Residues influencing rhesus Trim5α^TFP^ sensitivity surround a conserved capsid patch

**Figure 5:**
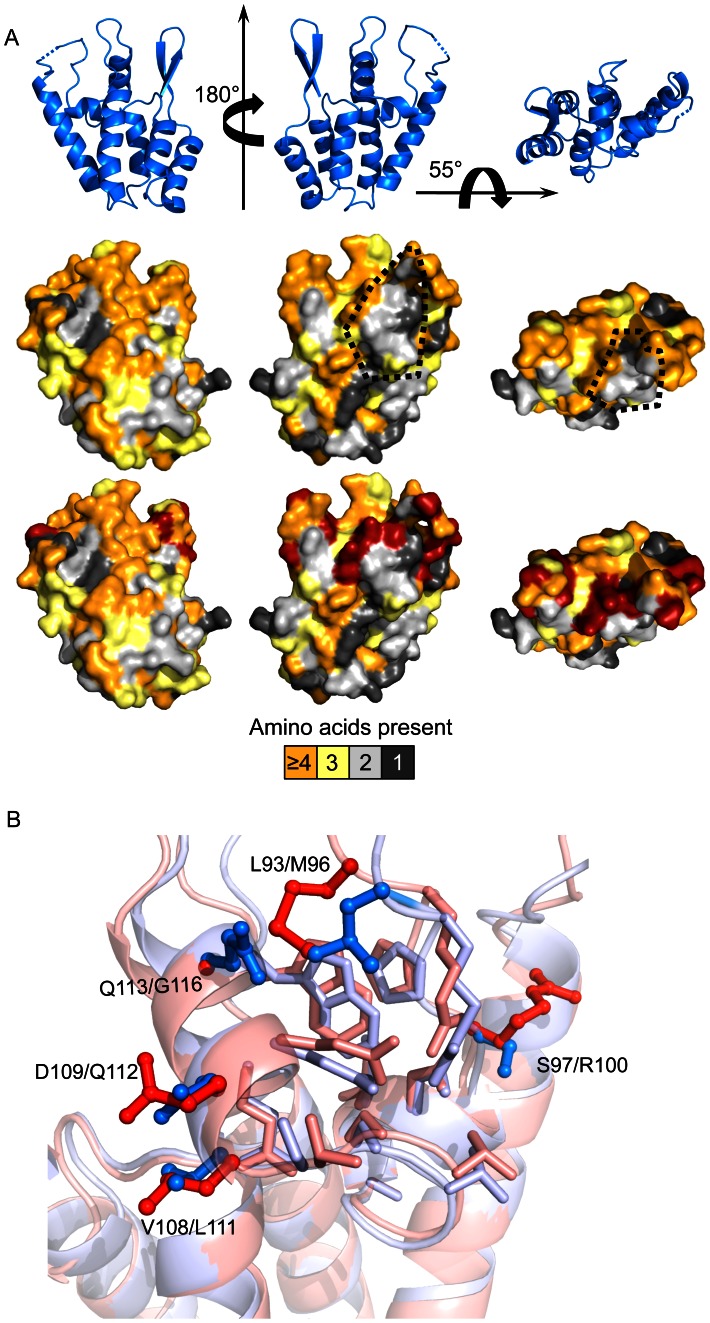
Mutations modulating rhesus Trim5α^TFP^ restriction ring a conserved surface patch. (A) Top row: Orientations of the SIVmac239 capsid used for Figure 5A. Middle row: Surface representation of the SIVmac239 capsid N-terminal domain colored to reflect amino acid conservation across divergent primate lentiviruses. The number of unique amino acids found at each position in an amino acid alignment of eleven divergent primate lentiviruses (Figure S7) was scored and colored according to the legend: Orange ≥4 unique amino acids at the specified position, yellow 3 unique amino acids at the specified position, light gray 2 unique residues at the specified position and dark gray 1 amino acid (100% conservation) at the specified position. The location of the conserved surface patch is indicated by dashed lines. Bottom panel: Locations of mutations that are associated with a >2.5 fold gain in sensitivity to rhTrim5α^TFP^ are shown in dark red. (B) Atomic view of the conserved surface patch. For reference the SIVmac239 and HIV-1 (2X2D) ribbon diagrams are shown in light blue and pink, respectively. The amino acids that make up the conserved surface patch are shown in sticks that are colored according to the capsid ribbon diagram, SIVmac239 in light blue and HIV-1 in light red. Variable positions shown to modulate rhesus Trim5α^TFP^ sensitivity are colored in dark blue (SIVmac239) and dark red sticks (HIV-1) for emphasis. Images created in PyMol.

In contrast to rhTrim5α^Q^, we found that rhTrim5α^TFP^ restricts at least three phylogenetically distinct primate lentiviruses: HIV-1, SIVagmTan, and SIVsm (Figure 1). While single amino acid substitutions affecting rhTrim5α^Q^ were confined to the *β*-hairpin, substitutions that increased sensitivity to rhTrim5α^TFP^ were spread across the N-terminal domain surface (Figures 4C and 4D). Based on these two observations, we hypothesized that rhTrim5α^TFP^ may have evolved to target a conserved element(s) unique to the primate lentivirus CA N-terminal domain. To identify uncharacterized sites of primate lentivirus conservation, we generated an alignment of CA N-terminal domains using one representative virus from eleven different primate lentivirus lineages (Figure S7). We then scored the number of unique amino acids found at each position, and mapped the results onto the SIVmac239 structure (Figure 5A).

Despite significant sequence diversity among primate lentiviruses, we found a cluster of conserved residues on the CA surface. This site overlapped with the structurally conserved C-terminus of the 4–5 loop, and helices 5 and 6. In SIVmac239, this patch is composed of residues Lue93, Arg94, Pro96, Gly98, Asp100, Ile101, Ala102, Gly103, Thr105, Ser106, Ser107, Glu110, Gln112 and Trp114 (Figures 5, S4, S5, and S7). This patch of conservation extends into a larger site of conservation formed by *α*-helices 3, 4 and 5. This site of conservation has recently been identified as the binding site for nuclear import factor CPSF6 [18]. Mutations that specifically increased sensitivity of SIVmac239 to rhTrim5α^TFP^ include S100R, V111L, D112Q and Q116G, which ring the boundaries of this patch, and Q86V, P87H, A89G, Δ91I, Q93P and L96M which are in the 4–5 loop just above the patch (Table 1, Figure 5 and Figure S5). In the immediate vicinity of the surface exposed conserved patch there were three observed trends for amino acid substitutions that influenced rhTrim5α^TFP^ restriction: 1) mutations in the variable regions of the 4–5 loop, 2) amino acid differences at the periphery of the surface patch, and 3) amino acid differences extending into the surface patch.

There were five amino acid substitutions within the highly variable regions of the 4–5 loop that had an impact on rhTrim5α^TFP^ restriction. The SIVmac239 4–5 loop, like that of HIV-2, is positioned further over the conserved surface patch than that of most HIV-1 loops. (Figures 4B and 5B). It has been documented that amino acid substitutions can alter the conformation or the dynamics of the 4–5 loop [77], [78]. It is therefore possible that Q86V, P87H, A89G, Δ91I and Q93P may alter the conformation or dynamics of the 4–5 loop in such a way as to enhance rhTrim5α recognition of the conserved surface patch.

Structurally, the surface patch was conserved across SIVmac239, HIV-1 and HIV-2. The C-terminus or the 4–5 loop, helix 5 and helix 6 were in very close agreement with the structures of HIV-1 and HIV-2, indicative of strong selection to preserve the overall architecture and amino acid composition of this site. Rather than changes to the structure or sequence of the patch, a majority of substitutions that altered rhTrim5α^TFP^ sensitivity were found at its periphery. For example, we found that altering Ser97 in SIVmac239 to the corresponding HIV-1 Arg had the largest effect of any single substitution tested. An Arg at this position is found in an overwhelming majority of reported SIVsm sequences, and importantly, the Arg to Ser mutation was found to be a critical adaptive change acquired by SIVsm to evade rhTrim5α^TFP^-mediated restriction *in vivo* [31]. In HIV-1 and HIV-2 an Arg at this position contributes to a hydrogen bond bridging the base of the 4–5 loop. In SIVmac239 the corresponding Ser97 does not participate in a similar contact, but rather, it appears to engage in additional contacts within helix 5 which are not observed in HIV-1 or HIV-2. SIVmac239 Gln109 and HIV-1 Asp112 are oriented similarly, however the presence of an acidic group would alter the chemical environment at the periphery of the patch (Figure 5B). There was no obvious difference to explain why the V111L mutant in helix-6 was six-fold more sensitive to restriction than parental SIVmac239. Perhaps slight differences between the side-chains of these residues can impact rhTrim5α^TFP^ restriction.

Two substitutions that were associated with increased rhTrim5α^TFP^ sensitivity extend into the conserved surface patch itself. We found that substituting the Leu at position 93 (which sits over the surface patch) for the less-bulky Met residue resulted in a 7-fold gain in sensitivity to rhTrim5α^TFP^ (Figure 5B and Table 1). Notably, Leu93/Met96 cover Trp114 and Arg94, both of which are absolutely conserved among primate lentiviruses. Finally, SIVmac239 residue Gln113 reaches deeper into the patch than the corresponding Gly116 in HIV-1nl4.3 (Figure 5B).

Together, mutagenesis and structural data suggests that rhTrim5α^TFP^ targets a surface-exposed patch of CA that is conserved in both structure and sequence across primate lentiviruses. Furthermore, differences between SIVmac239 and HIV-1 at the periphery of this patch account for their differential sensitivity to rhTrim5α^TFP^. At the same time, Trim5α^TFP^ and Trim5α^Q^ are both affected by changes in the *β*-hairpin, suggesting that restriction by both alleles involves recognition of this conserved feature of retroviral CAs.

### Evolution of Trim5α^TFP^

**Figure 6:**
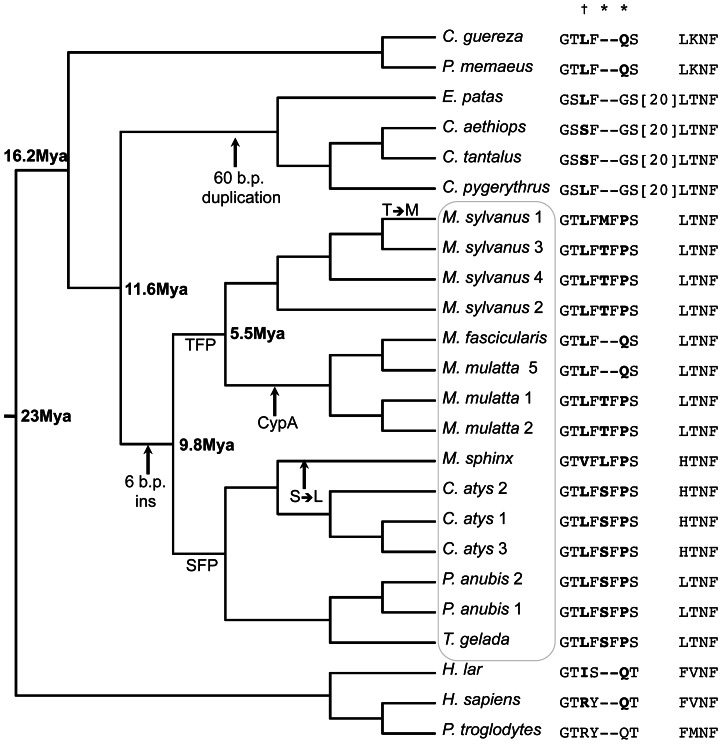
Evolutionary origins of the Trim5α^TFP^ allele. A Cladogram depicting the evolutionary relationships among Trim5 coding sequences from 16 extant primate species. Major divergence times are in bold, approximate dates of events discussed in the text are indicated with arrows. For each species/allele, the amino acid sequence corresponding to residues 335–346 (relative to rhesus Trim5) is shown; species names followed by numbers indicate multiple alleles. Residues with dN/dS >1 and a high posterior probability of positive selection are indicated by † (posterior probability >99%) or * (posterior probability >95%).

To reconstruct the evolutionary origins of the Q/TFP polymorphism, we analyzed multiple primate Trim5α sequences. We found that Gln341 in rhTrim5α is present at the homologous location in Trim5α of hominoids (*Homo sapiens* and *Pan troglodytes*), colobines (*C. guereza* and *P. nemaeus*) and macaques (*M. mulatta* and *M. fasicularis*) (Figure 6). In contrast, the insertion is found only in Papionins, including sooty mangabeys (*Cercocebus atys*), baboons (*P. anubis*), geladas (*T. gelada*), mandrills (*M. sphinx*), Barbary macaques (*M. sylvanus*), rhesus macaques (*M. mulatta*) and crab-eating macaques (*M. fasicularis*). Therefore, the insertion most likely originated in a common ancestor of the Papionini. Strikingly, a 60-nucleotide insertion/duplication at an identical position is found in Trim5 of cercopithecins (*E. patas* and other *Cercopithecus* species.). We therefore cannot rule out an earlier origin of the insertion in a common ancestor of the Cercopithecini and Papionini. Together, these observations give a range of insertion times between 9.8 and 11.6 million years ago (MYA) (Figure 6) [79]. Thus, Gln341 is the ancestral state at this position, and TFP is the evolutionarily derived state – consistent with our hypothesis that rhTrim5α^TFP^ alleles may be the result of selection to recognize the CA of primate lentiviruses.

We also noted considerable variation in the first codon of the inserted element itself, finding (in addition to TFP) orthologs encoding SFP, MFP and LFP among extant species (Figure 6). To ask whether this variation is consistent with continued positive selection since the time of insertion, we calculated dN/dS for each codon in the PRYSPRY domain using an alignment representing sixteen species of old world primate, including 4 species for which multiple haplotypes are available (*M. mulatta*, *M. sylvanus*, *P. anubis* and *C. atys*). We identified five codons in the PRYSPRY (332, 334, 337, 339 and 341) with high posterior probabilities of positive selection, including two in the 6 b.p. insertion itself (339 and 341), a pattern consistent with sequences evolving under continuous or repeated cycles of positive selection.

## Discussion

Rhesus macaques have three functionally distinct Trim5 alleles, rhTrim5α^TFP^, rhTrim5α^Q^, and rhTrim5^CypA^ [53], [54], [55], [58], [59], [60]. Of these, the structural basis for recognition of CA by rhTrim5^Cyp^ is best understood, and is attributed to interactions between the CypA domain and the 4–5 loop [65], [66]. In contrast, CA recognition by C-terminal PRYSPRY domains, such as those found in rhTrim5α^TFP^ and rhTrim5α^Q^, is not well understood. Using genetic, mutagenic, and structural approaches we found evidence that restriction by rhTrim5α proteins involves at least two structurally conserved elements of the primate lentivirus CA N-terminal domain.

There are four possible phenotypes for viruses that encounter rhTrim5α^TFP^ and rhTrim5α^Q^ alleles: resistance to both, sensitivity to both, and sensitivity to one or the other but not both. We observed only three of the four possibilities: resistance to both (SIVmac239), sensitivity to both (HIV-1nl4.3), and sensitivity to rhTrim5α^TFP^ but resistance to rhTrim5α^Q^ (SIVagmTAN, SIVsmE04, SIVsmE543 and HIV-2Rod) (Figure 1). We did not observe the converse, resistance to rhTrim5α^TFP^ combined with sensitivity to rhTrim5α^Q^. Moreover, none of the 34 chimeric viruses assayed displayed a rhTrim5α^TFP-res^/rhTrim5α^Q-sens^ phenotype, and there are no reports of other retroviruses displaying a rhTrim5α^TFP-res^/rhTrim5α^Q-sens^ phenotype. In fact, the only mutations in SIVmac239 that resulted in sensitivity to rhTrim5α^Q^ also resulted in sensitivity to rhTrim5α^TFP^ (Figures 3, S2 and Table 1).

The substitutions that increased sensitivity to both alleles map to the *β*-hairpin of CA. Structurally, the *β*-hairpin is the most conserved retroviral surface feature and is present in structures from five different genera [22], [23], [24], [25], [26], [27]. Thus, it appears that the *β*-hairpin is a retrovirus-associated molecular pattern by which Trim5α evolved to “recognize” retroviruses. In support of these hypotheses, we note that experimental evolution of a rhTrim5α^TFP^-resistant N-MLV in cell-culture selected for a single change in the *β*-hairpin of the MLV capsid [30]. When we superimposed the MLV and lentiviral CA structures, the identified resistance mutation in MLV overlaps with Y9, a residue we identified in the SIVmac239 *β*-hairpin that modulates recognition by rhTrim5α^TFP^ (Figure S8).

In addition to substitutions in the *β*-hairpin that increased sensitivity to both rhTrim5α^Q^ and rhTrim5α^TFP^, there were twelve additional mutations specifically associated with rhTrim5α^TFP^ restriction (Table 1). We interpret this to mean that the rhTrim5α^TFP^ allele has retained the CA-recognition capacity of rhTrim5α^Q^, but has evolved to interact with an additional target or targets in the lentiviral CA. These mutations map to surface features that distinguish primate lentivirus CAs from other retroviral CAs. Specifically, these substitutions ring a spatially clustered group of amino acids that are conserved across primate lentiviruses, altering this site at its periphery.

Interestingly, these mutations also overlap the binding sites of lentivirus-specific cellular cofactors, including CypA, NUP358 and CPSF6; notably, when these factors are fused to a Trim5 RBCC, the resulting fusion proteins function as restriction factors [18], [65], [66], [80], [81]. Primate lentiviruses have extended 4–5 loops that productively interact with at least two cellular cyclophilins, CypA and the CypA domain of a nuclear import factor, NUP358 [16], [17], [82]. In nature, these interactions have been independently exploited at least four times during primate evolution in the form of Trim5-CypA fusion proteins, two of which have been maintained in modern day lineages of owl monkeys and macaques [54], [55], [58], [59], [60], [83], [84], [85]. SIVmac239 residue Ala86 corresponds to Gly89 in the HIV-1 CypA binding motif, while SIVmac239 Gln88 and Gln89 are previously identified sites of an adaptive change permitting SIVmac to resist rhTrim5^CypA^ restriction [16], [31]. We demonstrate that both of these sites influence rhTrim5α^TFP^ restriction (Table 1). Resistance mutations to both rhTrim5^CypA^ and rhTrim5α^TFP^ may explain why SIVmac239 does not utilize Nup358, which is required by other primate lentivirusess for efficient nuclear import and optimal target site integration [17].

The conserved surface patch is an extension of the CPSF6 binding site, which is conserved among primate lentiviruses [18]. Our data suggest that this site is targeted by the rhTrim5α^TFP^ PRYSPRY domain (Figure 5). We therefore propose that the targeting of this site is analogous to exploitation of the CypA binding site in the 4–5loop by rhTrim5^CypA^, since rhTrim5α^TFP^ also exploits a critical, conserved CA interface that is necessary for its interaction with a host co-factor that facilitates lentiviral replication.

Recent structural determination of the rhesusTrim5α PRYSPRY domain shows the four discrete variable regions are arranged on the surface of a *β*-sandwich core [71], [72]. Ohkura *et al.* reported that the variable regions may make independent contributions to CA recognition [86]. Thus, differences in targeting by the rhTrim5α^Q^ and rhTrim5α^TFP^ proteins may reflect contributions from different regions of the PRYSPRY domain. For example, the TFP insertion in variable region 1 (V1) may directly confer specificity for the conserved face of lentiviral CAs, whereas the interactions of both rhTrim5α^TFP^ and rhTrim5α^Q^ with the *β*-hairpin may involve contributions from one or more of the other variable loops.

The original insertion in V1 that gave rise to rhTrim5^TFP^ in modern macaques arose after the *Cercopithecinae*-*Colobinae* split, but prior to divergence of the *Macaca* and *Papio* lineages, providing an estimate for the time of insertion between 9.8 to 11.6 million years ago [79]. In contrast, the Trim5^CypA^ allele has only been found in Asian macaques, but not in Barbary macaques or any other old world primates [54], [55], [58], [59], [60], [87], and may therefore have arisen less than 5–6 million years ago, after the lineage leading to Asian macaques (*Macaca sp.*) diverged from the African lineages [79]. These dates, and the observation that rhTrim5α^TFP^ and rhTrim5^CypA^ target lentiviral-specific features of CA, constitute indirect but compelling evidence that viruses related to modern primate lentiviruses were infecting ancestral primates as far back as 12 million years ago, driving selection of Trim5 variants with enhanced capacity to restrict lentiviral replication. Recently, similar conclusions were independently obtained from a study of APOBEC3G variation in Old World monkeys [88]. Endogenous lentiviral sequences found in the genomes of European brown rabbits [89], Malagasey lemurs [90] and weasels [91], [92] support the conclusion that lentiviruses were extant at this time, and structural studies indicate that the CA proteins of at least two of these (RELIK and pSIVgml) were very similar to modern lentiviruses [93].

**Figure 7:**
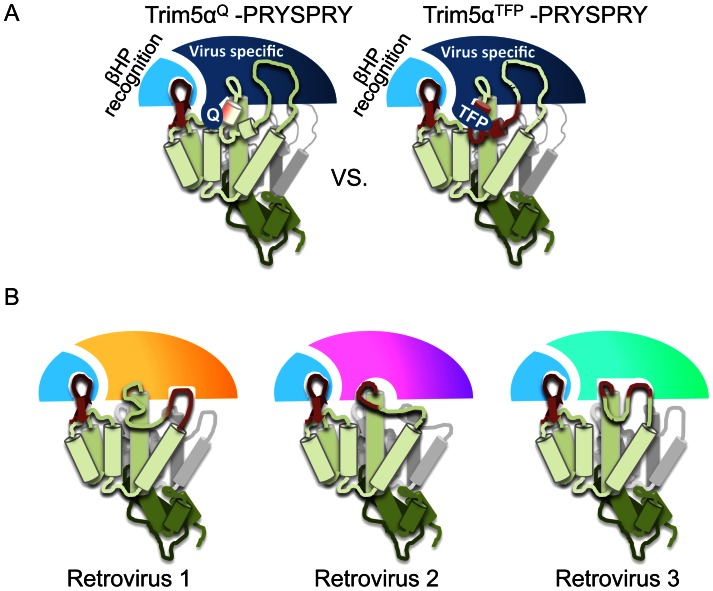
A Proposed model for the evolution of novel Trim5α variants. The rhTrim5α^Q^ alleles and the rhTrim5α^TFP^ alleles share the ability to recognize lentiviral *β-*hairpins. The rhTrim5α^TFP^ alleles evolved to recognize the conserved surface patch. We believe this observation underscores an inherent uncoupling between capsid recognition modules within the PRYSPRY domain. The *β-*hairpin is a conserved feature found in all reported retroviral capsid structures, and therefore a convenient target for host proteins that evolve to recognize a broad range of retroviruses. We believe *β-*hairpin targeting is a conserved feature of Trim5α proteins and allows for the evolution of specificity of capsid recognition. (A) Evolution of conserved surface patch recognition. The rhTrim5α^Q^ allele is capable of strongly recognizing the *β-*hairpin (dark red) and able to engage in a weaker contact (pink) with helix 6, at one edge of the conserved surface patch. Recognition of these two features is conserved between rhTrim5α^Q^ and rhTrim5α^TFP^ alleles and therefore unaffected by the Q/TFP polymorphism. The region of the PRYSPRY that encodes for the intrinsic *β-*hairpin recognition module is colored light blue and the module that can adapt to specific viruses is colored dark blue. We propose that the polymorphic region of variable loop 1 (V1) is uncoupled from intrinsic *β-*hairpin recognition (by of another region within the PRYSPRY) allowing it to tolerate mutations such as the 6 nucleotide insertion. In this model, the rhTrim5α^TFP^ allele engages in similar contacts as the rhTrim5α^Q^ allele, but has gained the ability to target the conserved surface patch (dark red). (B) The ability to recognize a conserved retroviral element, even if it allows very weak associations retroviral capsids, can allow for the selection of additional capsid binding modules within the PRYSPRY domain allowing it to adapt to specific retroviral pressures. Hypothetical adaptation to different retroviral targets are depicted as differently colored PRYSPRY domains. Together this process could lead to the breadth and specificity observed among Trim5α orthologs. For simplicity this model is depicted with one PRYSPRY domain recognizing one capsid monomer, although the stoichiometry or orientation of Trim5α binding is not known at this time.

The natural history of African primate lentiviruses, and the species that harbor them, suggests lentiviruses were a driving force for the selection and maintenance of TFP-like Trim5α alleles during the last 12 million years. Based on these observations, we propose an evolutionary model in which different regions of the PRYSPRY can evolve independently to recognize different features of retroviral CAs (Figure 7). *β*-hairpin recognition was conserved between the ancestral Trim5α^Q^ allele and the evolutionary derived rhTrim5α^TFP^ allele. Therefore, it is likely that the region encompassing the Q/TFP polymorphism in variable loop 1 (V1) does not contribute to *β*-hairpin recognition. Instead, this region may be free to make additional contacts with the CA. Due to its dynamic and unstructured nature, V1 may readily tolerate mutations and insertions (such as the 6-nucleotide insertion) affording the molecule enhanced evolutionary plasticity [71], [72]. The SIV-HIV_h6_ mutant was restricted by rhTrim5α^Q^, implying that the rhTrim5α^Q^ PRYSPRY could recognize one edge of the conserved surface patch (Figure 7A). The modern day presence of Trim5α orthologs with the 6-nucleotide insertion indicate that the insertion event conferred a selective advantage (likely against primate lentiviruses). The simplest explanation is that the insertion allowed V1 to make additional contacts or possibly even extend beyond helix 6 and further into the conserved surface patch. We have shown that the first and last positions of the rhesus TFP polymorphism have been under positive selection, indicative of continued refinement of its ability to recognize the conserved surface patch over evolutionary time.

This model is likely a snapshot of a larger evolutionary scenario in which an ancestral PRYSPRY domain may first have acquired the ability to recognize a highly conserved retroviral CA element (such as the *β*-hairpin). On top of this intrinsic recognition ability, modularity of Trim5α proteins allowed them to explore additional targets on the CA surface in response to pressures from specific viruses or viral families, perhaps by taking advantage of inherent plasticity within the variable loops (Figure 7B). Such a process, played out over the course of tens of millions of years of evolution, could help to explain both the collective breadth and species-specificity of modern primate Trim5α proteins.

## Methods

### Cell lines

Crandell-Rees Feline Kidney (CRFK) cells and Human Embryonic Kidney 293T/17 (HEK293T/17) cells were obtained from American Type Culture Collection (Manassas, VA) and grown in DMEM/10% FBS. CRFK cell lines stably expressing N-terminally HA-tagged Trim5 orthologs were previously described [31]. Stable cell lines were maintained in DMEM/10% FBS supplemented with 0.5 mg/ml G418. All cultured cells were maintained at 37°C with 5% CO2.

### Plasmids and mutagenesis

The SIVmac239-based retroviral vector pV1EGFP (gift from Hung Fan, University of California, Irvine, CA) was previously modified to contain a functional gag-pol ORF [31].

All single cycle chimeric viruses are in either the pV1EGFP-SIV or HIV-1nl4.3 pNL43DenvGFP background as indicated. To facilitate the rapid production of chimeric viruses, a capsid and gag shuttle vector system was engineered through DNA synthesis by GENEART (Regensburg, Germany). Silent nucleotide changes within the capsid allowed for chimerization between capsids from either virus (Figure S9). All chimeric capsids with the exception of single amino acid point mutants were produced through gene synthesis by GENEART (Regensburg, Germany) and were then cloned into the proper viruses using our shuttle vector system. Single amino acid substitutions on the SIVmac239 surface were made using site directed mutagenesis. The S100R mutant was described in a previous publication [31].

A CFP expressing HIV-1 derived lentiviral vector was created for abrogation assays. A CFP gene was introduced into using AgeI an XhoI sites into pNL-EGFP/CMV-WPREDU3 [6], a vector based on pNL-EGFP/CMV (which features the WPRE element for increased mRNA stability and a deleted U3 region for added safety).

### Virus production

All single-cycle viruses were produced in HEK293T/17 cells by cotransfection of the appropriate viral plasmid and pVSV-G (Clontech Laboratories, Mountain View, CA), using the GenJet transfection system (SignaGen; Ijamsville, MD). Culture supernatants containing the single-cycle, GFP/EGFP expressing, VSV-G-pseudotyped virions were titered on untransfected CRFK cells; supernatant volumes resulting in approximately 25% GFP/EGFP+ CRFK cells were used for infectivity assays on the cell lines expressing the indicated Trim5α. Information regarding viral infectivity appears in Figure S2.

The CFP expressing HIV-1 lentiviral vector was made from 293T transfection of a 3∶2∶1 plasmid ratio of pNL-ECFP/CMV-WPREDU3 [6], pCD/NL-BH*DDD [94] and pVSV-G (Clontech Laboratories, Mountain View, CA) (pNL-ECFP/CMV-WPREDU3 and pCD/NL-BH*DDD were kindly provided by Dr. Jakob Reiser, Louisiana State University Health Sciences Center).

### Infectivity assays

Stably expressing Trim5 CRFK cells were seeded at a concentration of 5×10^4^ cells per well in 12-well-plates and infected with the appropriate amount of VSV-G pseudotyped, single-cycle, GFP/EGFP expressing viruses. All infections were done in triplicate. After 2 days, expression of GFP/EGFP was analyzed by fluorescence-activated cell sorting (FACS) performed on a FACSCaliburTM flow cytometer (BD, Franklin Lakes, NJ), and data were analyzed using FlowJo software (Tree Star, Inc., Ashland, OR). Viral titers were determined using the appropriate p24 (HIV-1) or p27 (SIVmac) antigen capture kit from Advanced Bioscience Labs (Rockville, MD). Information regarding viral titers appears in Figure S2.

### Protein expression and purification

A codon optimized N-terminal fragment of the SIVmac239 capsid corresponding to residues 1–144 was synthesized with a C-terminal factor Xa cleavage site and 6x-His Tag by GENEART (Regensburg, Germany). Using engineered *XbaI* and *XhoI* sites the N-terminal fragment was cloned into pET303 (Invitrogen) and expressed from BL21(DE3) *E. coli* cells. The SIVmac239 capsid was purified by Ni-NTA agarose (Qiagen) followed by gel filtration chromatography on a Superdex 200 column (GE Healthcare). The C-terminal 6x-His tag was removed by treatment with factor Xa (New England Biolabs), re-purified by orthogonal Ni-NTA agarose chromatography and gel filtration chromatography.

### Crystallization

Purified SIVmac239 capsid protein was crystallized by the hanging drop method over a reservoir solution containing 10%(w/v) PEG 2000 MME, 10 mM nickel chloride and 100 mM TRIS, pH 8.5 at 24 C. Crystals were harvested from 0.2 ul drops and cryoprotected by addition of 10–15% PEG 400 or glycerol to the reservoir solution, then flash cooled in liquid nitrogen. Protein concentration ranged from 10–15 mg/ml.

### Structure determination and refinement

We recorded diffraction data at beamline 24-ID-E at the Advanced Photon Source. Data sets from individual crystals were processed with HKL2000 [95]. Molecular replacement (MR) was carried out with PHASER [96] using the HIV-2 capsid as an initial search model. One molecule of SIVmac239 completes the asymmetric unit. Refinement was carried out using PHENIX [97], [98] and all model modifications were done in COOT [99]. Initial rigid body refinement followed by simulated annealing and positional refinement was done. The 4–5 loop (residues 83–97) was initially removed from the model and rebuilt into modest density. There was no clear density for residue proline 88 and it was omitted from the structure. The model was further refined by additional cycles of positional and B-factor refinement, followed by TLS. The quality of the data was assessed using MolProbity [100]. Data collection and refinement statistics can be found in Table S1. Coordinates and diffraction data have been submitted to the PDB, accession number: PDB:4HTW.

### Sequence analysis

Trim5α sequences were identified by BLAST search of the non-redundant nucleotide database, aligned in Geneious Pro v.5.5.4 using the Translation Align option. The alignment was adjusted manually, converted back to nucleotide and the best-fit tree identified with MrBayes. dN/dS analysis was performed with CODEML in v4.4 of PAML (Table S2) [101].

## Supporting Information

Figure S1 **Amino acid alignment of chimeric viruses.** Amino acid sequences of chimeric viruses used in this manuscript aligned to SIVmac239. Black lettering indicates unique SIVmac239 amino acids. Red lettering indicates unique HIV-1nl4.3 amino acids. Gray dots indicate conserved positions between SIVmac239 and HIV-1nl4.3. Hyphens were inserted to preserve the alignment in cases of insertions/deletions. Numbered rows correspond to the following viruses: 1. SIV-HIV_Interior_ 2. SIV-HIV_surface_ 3. SIV-HIV_bhp_ 4. SIV-HIV_bhpQ7Δ_, 5. SIV-HIV_4–5L_ 6. SIV-HIV_h6_ 7. HIV-SIV_surface_ 8. HIV-SIV_surface25_ 9. SIV_V2I_10. SIV_Q3V_ 11. SIV_I5N_ 12. SIV_G6L_ 13. SIV_Δ7Q_ 14. SIV_N9Q_ 15. SIV_Y10M_ 16. SIV_Q86V_ 17. SIV_P87H_ 18. SIV_Δ88A_ 19. SIV_A89G_ 20. SIV_Δ91I_ 21. SIV_Q92A_ 22. SIV_Q93P_ 23. SIV_L96M_ 24. SIV_S100R_ 25. SIV_S110T_ 26. SIV_V111L_ 27. SIV_D112Q_ 28. SIV_Q116G_ 29. SIV_Y119T_ 30. SIV_R120H_ 31. SIV_R120N_ 32. SIV_Q121Δ_ 33. SIV_Q122N_ 34. SIV_N123P_
(TIF)

Figure S2 **Characterization of viruses.** The titers and infectivities of viruses presented in this manuscript are provided. Titers were determined by p24 and p27 antigen capture ELISA (Advanced Bioscience Laboratories, Rockville MD.). All viruses in which the C-terminal domain was derived from HIV-1 were used with p24 antigen capture kit, while all viruses in which the C-terminal domain was derived from SIVmac239 were tested using a p27 antigen capture kit.
(TIF)

Figure S3 **Surface feature chimeras do not abrogate Trim5α activity.** Two independent saturation controls were done to insure that attenuated viruses did not abrogate Trim5α activity. (A) Titration curves on CRFK-Neo control cells (Black lines I–IV) and mamu1 (rhTrim5α^TFP^) expression cells (red lines I–IV) were carried out. Data points are the average of 3 infections. Error bars indicate the S.E.M. 50,000 cells were seeded in a 24 well plate in 0.5 ml of media. Infections were carried out in 0.2 ml media and harvested for FACS 40 hours post infection. (I) SIVmac239. (II) HIV-1nl4.3. (III) HIV-SIV_surface_. (IV) HIV-SIV_surface25_. Notably there is little or no deviation between the apparent infectivities of SIVmac239, HIV-SIV_surface_ and HIV-SIV_surface25_ on control cells and on mamu1 expressing cells at every concentration of virus tested. There is a very large difference between the apparent infectivity of HIV-1nl4.3 on control cells and mamu1 (rhTrim5α^TFP^) cells. (V) Graphs I–IV graphed together. Importantly, despite the attenuation of HIV-SIV_surface_ and HIV-SIV_surface25_ their curves fall inside the saturating curve for HIV-1nl4.3 on mamu1 cells. (B) Two color abrogation assays were conducted under identical conditions to those in Table 1 and Figures 1 and 3. Cells were harvested at 30 hours post infection. Identical amounts of HIV-1, SIVmac239-S100R, HIV-SIV_surface_ and HIV-SIV_surface25_ to those used in Figure 3 and Table 1 were used. Additionally, the same concentration (ng of capsid) as the most attenuated mutant, HIV-SIV_surface_, was used for the two rhTrim5α restricted viruses HIV-1nl4.3 and SIVmac239 S100R. Cells were co-infected with a fixed concentration of a HIV-1 CFP reporter virus. Values for GFP and CFP positive cells are separated into two columns (“GFP” and “CFP”) for ease of viewing, but the values are from the same co-infection. Under all conditions an enhancement of infectivity for the CFP reporter virus on restrictive cells mamu1 and mamu4 (rhTrim5α^TFP^ and rhTrim5α^Q^) was not observed. Therefore, despite high concentrations of virus, our experimental conditions did not saturate Trim5α. Bar graphs represent the average of 3 independent infections. Error bars indicate the S.E.M.
(TIF)

Figure S4 **B-factor Analysis of SIVmac239 structure.** (A) Average B-factor plot of each residue included in the final model. The *β*-hairpin and 4–5 loop are delineated as reference points. (B) Visual “heat-map” of average B-factors. Residue 88 was removed from the structure due to lack of clear density and is indicated by the dashed red line. Images created in PyMol.
(TIF)

Figure S5 **Electron Density Maps of Key Regions in the SIVmac239 structure.** (A) the *β*-hairpin, residues 1–14. (B) the CypA binding loop, residues 83–100—residue 88 has been removed from the structure as there was no clear electron density (C) the “conserved patch” residues 95–116 (D) isolated residues 94–106 and (E) 106–116. All images are 2Fo-Fc maps and are contoured at 1.5**σ** throughout for consistency. Structure factors and the final model have been deposited in the Protein Data Bank accession 4HTW. All images created in PyMol
(TIF)

Figure S6 **Mutations modulating Trim5α sensitivity mapped to the HIV-1 hexamer.** Mutations from Table 1 mapped to the HIV-1 hexamer structure 3GV2. Restriction data for mutant viruses tested against the rhesus Trim5α^TFP^ allele mamu1 (A) and the rhesus Trim5α^Q^ allele mamu4 (B). Positions that were mutated on the capsid surface and were <2.5 fold more sensitive to Trim5α restriction than SIVmac239 are shown in gray spheres Orange spheres show the location of mutations associated with 2.5–5 fold gains in sensitivity to rhesus Trim5α. Red spheres indicate positions associated with >5 fold gains in sensitivity to rhesus Trim5α. Images created in PyMol
(TIF)

Figure S7 **Amino acid alignment of divergent primate lentiviruses.** Primate lentiviruses from eleven different lineages are aligned corresponding to the published alignment found in the Los Alamos Sequence database. Accession numbers: SIVmac239-M33262, HIV-1-K03455, SIVcol-AF301156, SIVlho-AF075269, SIVagm-U58991, SIVgsn-AF468658, SIVwrc-AM745105, SIVdrl-AY159321, SIVmand2-AY159322, SIVdeb-AY523865, SIVtal-AM182197
(TIF)

Figure S8 **Structural comparison between SIVmac239 and MLVs with differential restriction by rhesus Trim5α.** (A) *β*-hairpin of N-Tropic MLV (PDB: 1U7K) with residue L10 shown in sticks and spheres (B) *β*-hairpin of the N-MLV L10W mutant (PDB:2Y4Z) that is rhesus Trim5α^TFP^ resistant, 10W shown in sticks and spheres. (C) *β*-hairpin of HIV-1 (PDB:2X2D) with M10 shown in sticks and spheres. (D) SIVmac239 *β*-hairpin Y9 shown in sticks and spheres. (E) Structural alignment of rhesus Trim5α sensitive N-MLV with HIV-1. (F) Structural alignment of the rhesus Trim5α resistant N-MLV L10W with SIVmac239. Images created in PyMol
(TIF)

Figure S9 **Schematic of synthesized genes and cloning strategy used to generate chimeric viruses.** All constructs were synthesized by GENEART (Regensburg, Germany). Numbering corresponds to the standard HXB2 and SIVmac239 numbering, respectively. For efficient exchange of capsids between viruses and chimerization within capsids silent nucleotide changes were made in both viruses creating identical restriction sites. Naturally occurring restriction sites at the ends of the shuttle vector are used for insertion into the proper parental virus. Amino acid differences at the N-terminus of the CA protein did not allow us to use a single common enzyme for this site. Instead SIVmac239 constructs use a BsrGI site while HIV-1nl4.3 constructs use an MfeI site. Two additional shuttle vectors were made to accommodate either N-terminus in both SIVmac239 and HIV-1nl4.3 backbones.
(TIF)

Table S1 **Crystallography refinement statistics.**
(PDF)
