# Supplementary figures and images for: Gain-of-Sensitivity Mutations in a Trim5-Resistant Primary Isolate of Pathogenic SIV Identify Two Independent Conserved Determinants of Trim5α Specificity
Source: PLoS Pathog. 2013 May 9;9(5):e1003352. doi: 10.1371/journal.ppat.1003352 (PMC3649984; doi:10.1371/journal.ppat.1003352)

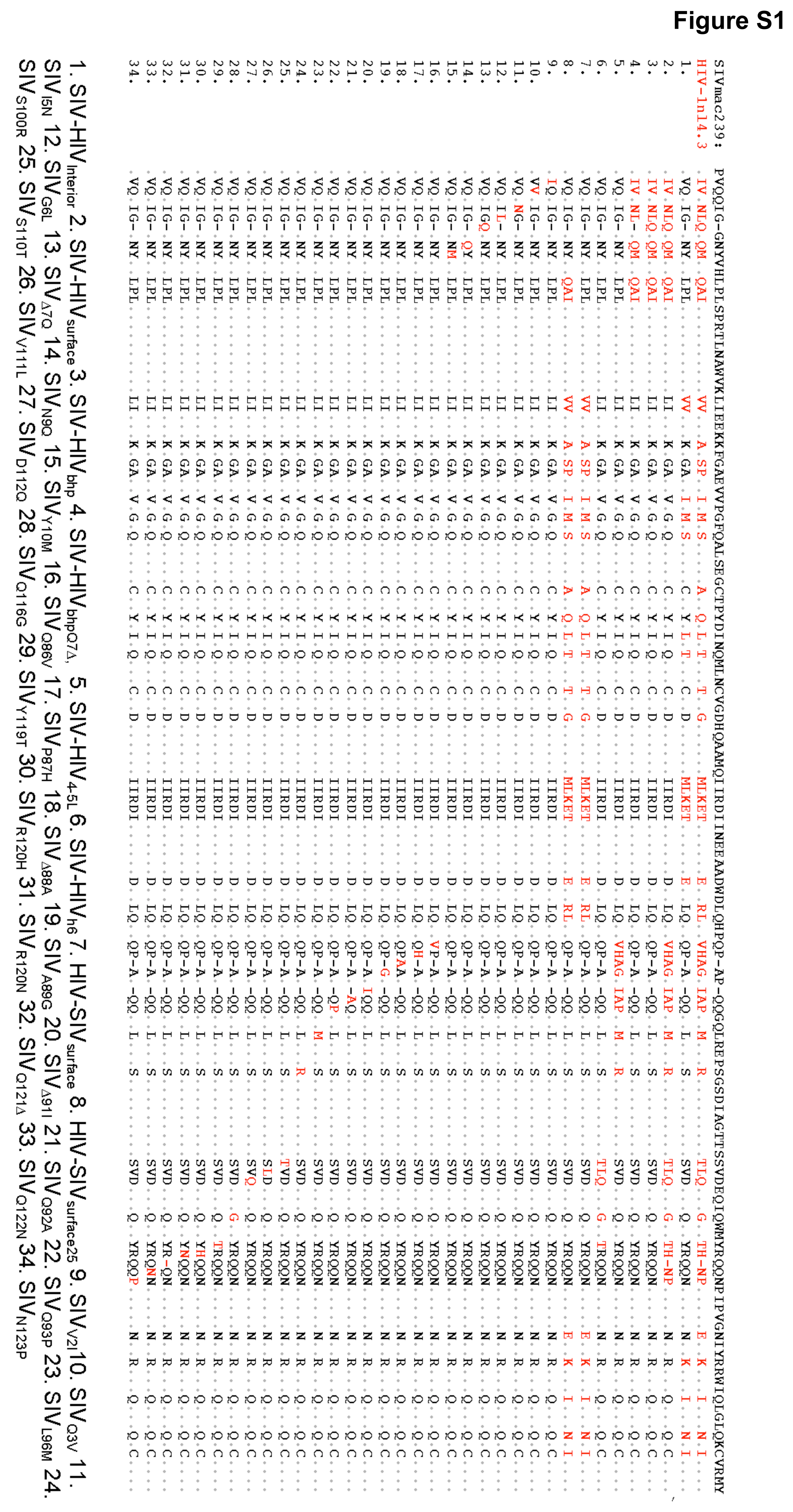

Supplement: Figure S1 — Amino acid alignment of chimeric viruses. Amino acid sequences of chimeric viruses used in this manuscript aligned to SIVmac239. Black lettering indicates unique SIVmac239 amino acids. Red lettering indicates unique HIV-1nl4.3 amino acids. Gray dots indicate conserved positions between SIVmac239 and HIV-1nl4.3. Hyphens were inserted to preserve the alignment in cases of insertions/deletions. Numbered rows correspond to the following viruses: 1. SIV-HIVInterior 2. SIV-HIVsurface 3. SIV-HIVbhp 4. SIV-HIVbhpQ7Δ, 5. SIV-HIV4–5L 6. SIV-HIVh6 7. HIV-SIVsurface 8. HIV-SIVsurface25 9. SIVV2I10. SIVQ3V 11. SIVI5N 12. SIVG6L 13. SIVΔ7Q 14. SIVN9Q 15. SIVY10M 16. SIVQ86V 17. SIVP87H 18. SIVΔ88A 19. SIVA89G 20. SIVΔ91I 21. SIVQ92A 22. SIVQ93P 23. SIVL96M 24. SIVS100R 25. SIVS110T 26. SIVV111L 27. SIVD112Q 28. SIVQ116G 29. SIVY119T 30. SIVR120H 31. SIVR120N 32. SIVQ121Δ 33. SIVQ122N 34. SIVN123P (TIF) [file ppat.1003352.s001.tif]

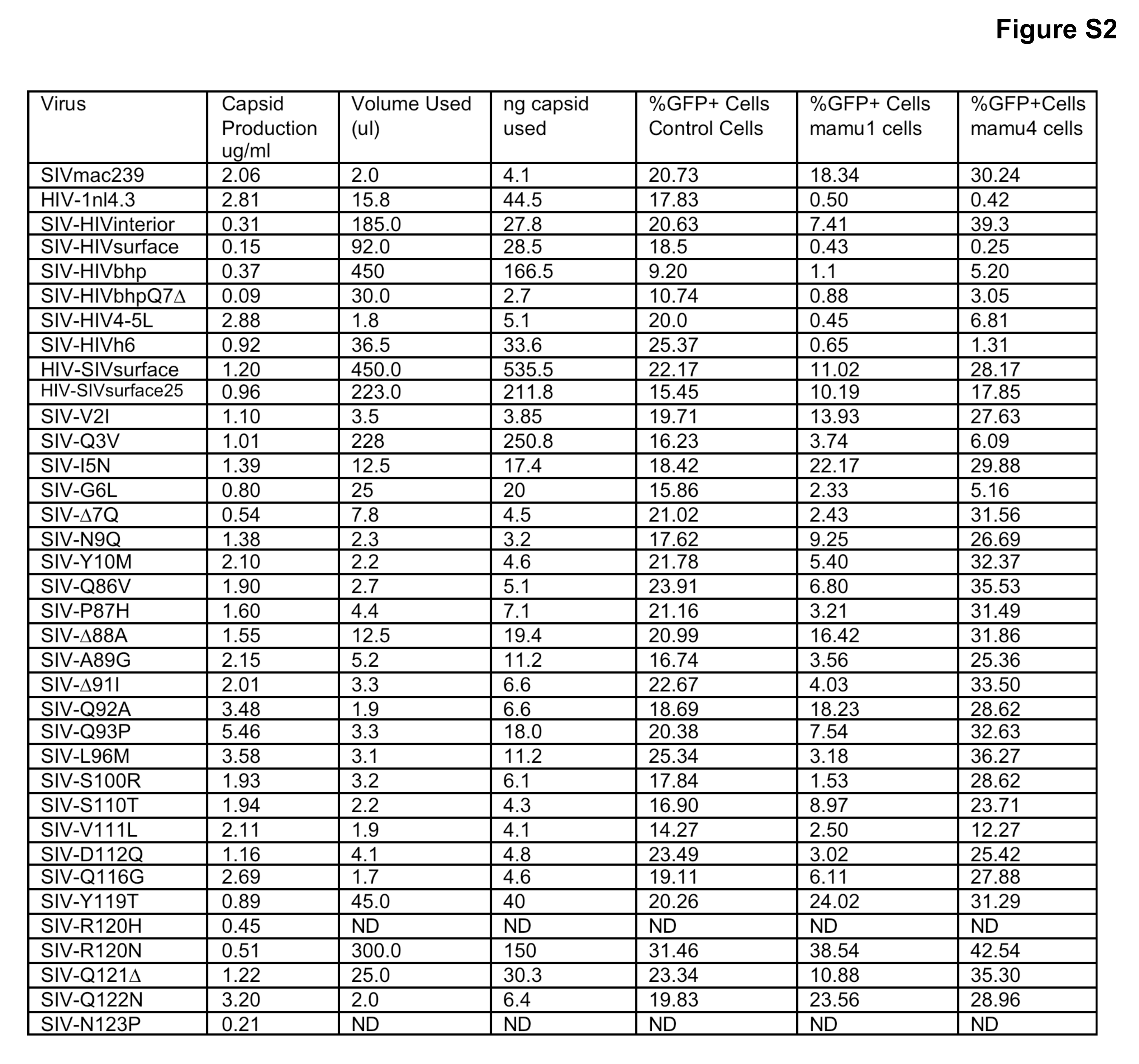

Supplement: Figure S2 — Characterization of viruses. The titers and infectivities of viruses presented in this manuscript are provided. Titers were determined by p24 and p27 antigen capture ELISA (Advanced Bioscience Laboratories, Rockville MD.). All viruses in which the C-terminal domain was derived from HIV-1 were used with p24 antigen capture kit, while all viruses in which the C-terminal domain was derived from SIVmac239 were tested using a p27 antigen capture kit. (TIF) [file ppat.1003352.s002.tif]

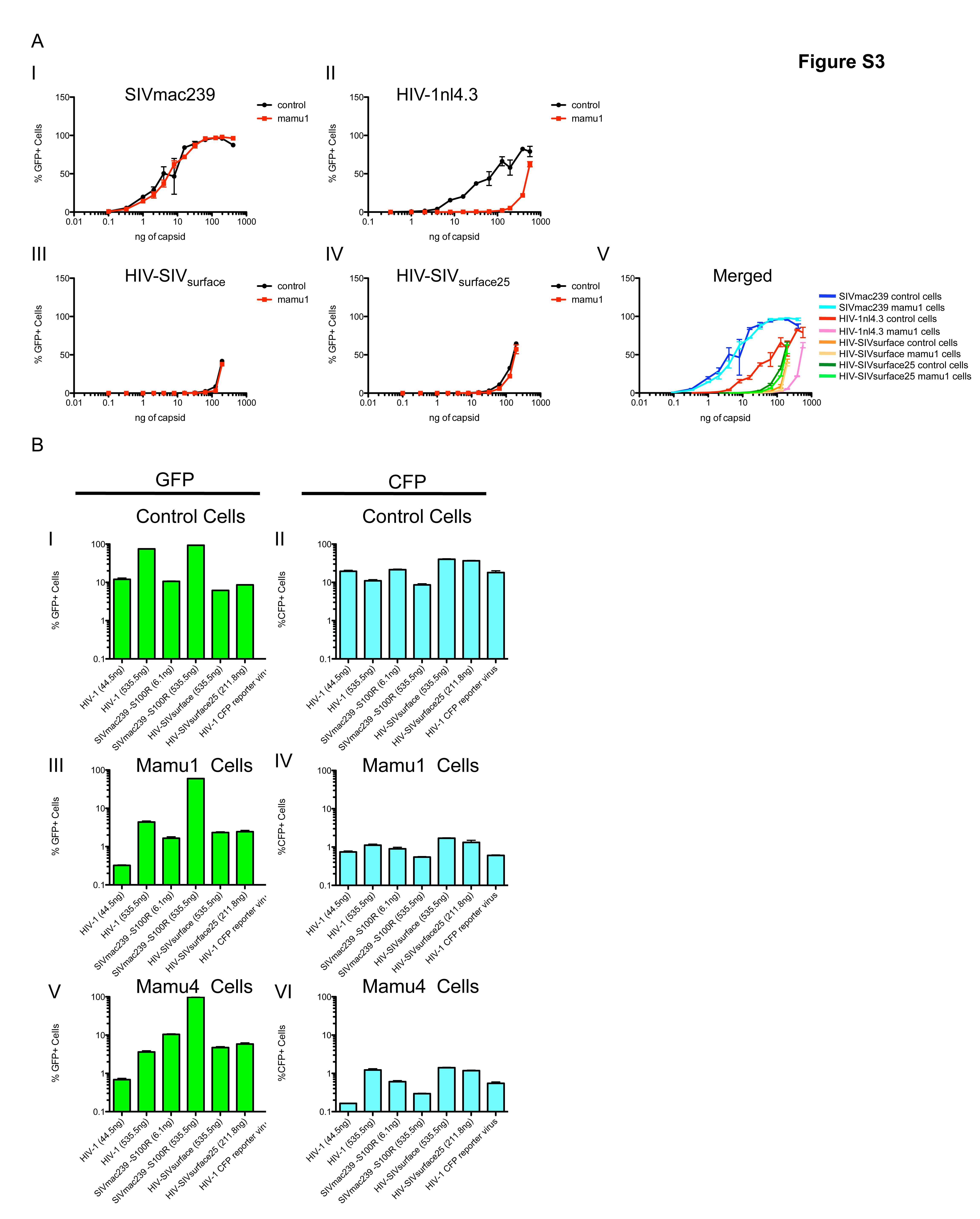

Supplement: Figure S3 — Surface feature chimeras do not abrogate Trim5α activity. Two independent saturation controls were done to insure that attenuated viruses did not abrogate Trim5α activity. (A) Titration curves on CRFK-Neo control cells (Black lines I–IV) and mamu1 (rhTrim5αTFP) expression cells (red lines I–IV) were carried out. Data points are the average of 3 infections. Error bars indicate the S.E.M. 50,000 cells were seeded in a 24 well plate in 0.5 ml of media. Infections were carried out in 0.2 ml media and harvested for FACS 40 hours post infection. (I) SIVmac239. (II) HIV-1nl4.3. (III) HIV-SIVsurface. (IV) HIV-SIVsurface25. Notably there is little or no deviation between the apparent infectivities of SIVmac239, HIV-SIVsurface and HIV-SIVsurface25 on control cells and on mamu1 expressing cells at every concentration of virus tested. There is a very large difference between the apparent infectivity of HIV-1nl4.3 on control cells and mamu1 (rhTrim5αTFP) cells. (V) Graphs I–IV graphed together. Importantly, despite the attenuation of HIV-SIVsurface and HIV-SIVsurface25 their curves fall inside the saturating curve for HIV-1nl4.3 on mamu1 cells. (B) Two color abrogation assays were conducted under identical conditions to those in Table 1 and Figures 1 and 3. Cells were harvested at 30 hours post infection. Identical amounts of HIV-1, SIVmac239-S100R, HIV-SIVsurface and HIV-SIVsurface25 to those used in Figure 3 and Table 1 were used. Additionally, the same concentration (ng of capsid) as the most attenuated mutant, HIV-SIVsurface, was used for the two rhTrim5α restricted viruses HIV-1nl4.3 and SIVmac239 S100R. Cells were co-infected with a fixed concentration of a HIV-1 CFP reporter virus. Values for GFP and CFP positive cells are separated into two columns (“GFP” and “CFP”) for ease of viewing, but the values are from the same co-infection. Under all conditions an enhancement of infectivity for the CFP reporter virus on restrictive cells mamu1 and mamu4 (rhTrim5αTFP and rhTrim5α [file ppat.1003352.s003.tif]

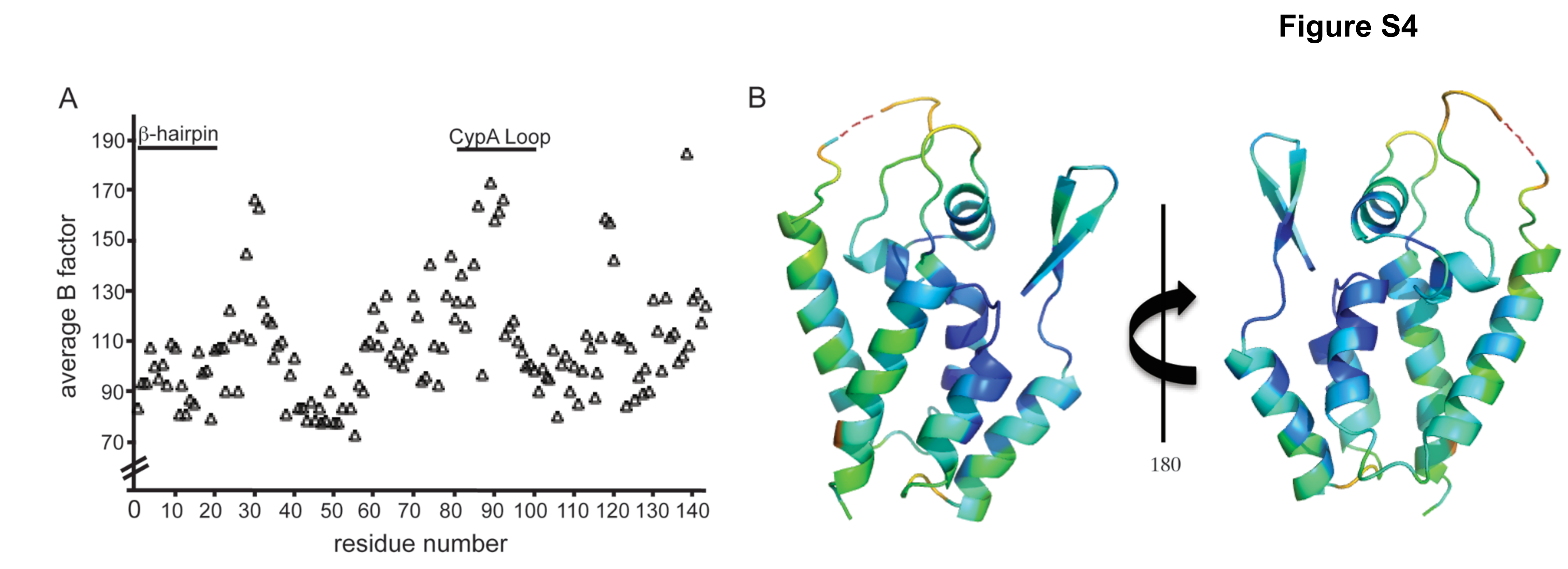

Supplement: Figure S4 — B-factor Analysis of SIVmac239 structure. (A) Average B-factor plot of each residue included in the final model. The β-hairpin and 4–5 loop are delineated as reference points. (B) Visual “heat-map” of average B-factors. Residue 88 was removed from the structure due to lack of clear density and is indicated by the dashed red line. Images created in PyMol. (TIF) [file ppat.1003352.s004.tif]

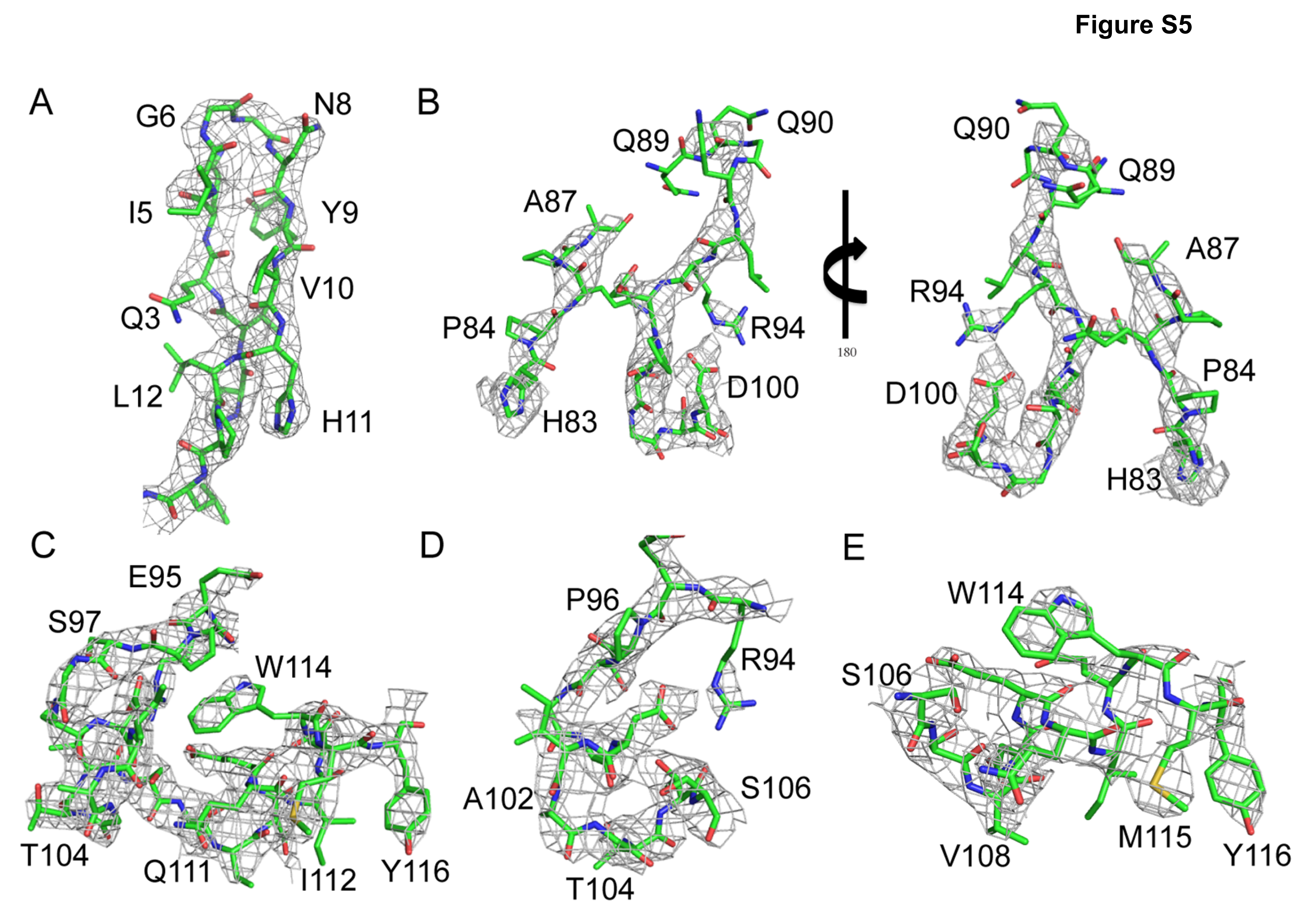

Supplement: Figure S5 — Electron Density Maps of Key Regions in the SIVmac239 structure. (A) the β-hairpin, residues 1–14. (B) the CypA binding loop, residues 83–100—residue 88 has been removed from the structure as there was no clear electron density (C) the “conserved patch” residues 95–116 (D) isolated residues 94–106 and (E) 106–116. All images are 2Fo-Fc maps and are contoured at 1.5σ throughout for consistency. Structure factors and the final model have been deposited in the Protein Data Bank accession 4HTW. All images created in PyMol (TIF) [file ppat.1003352.s005.tif]

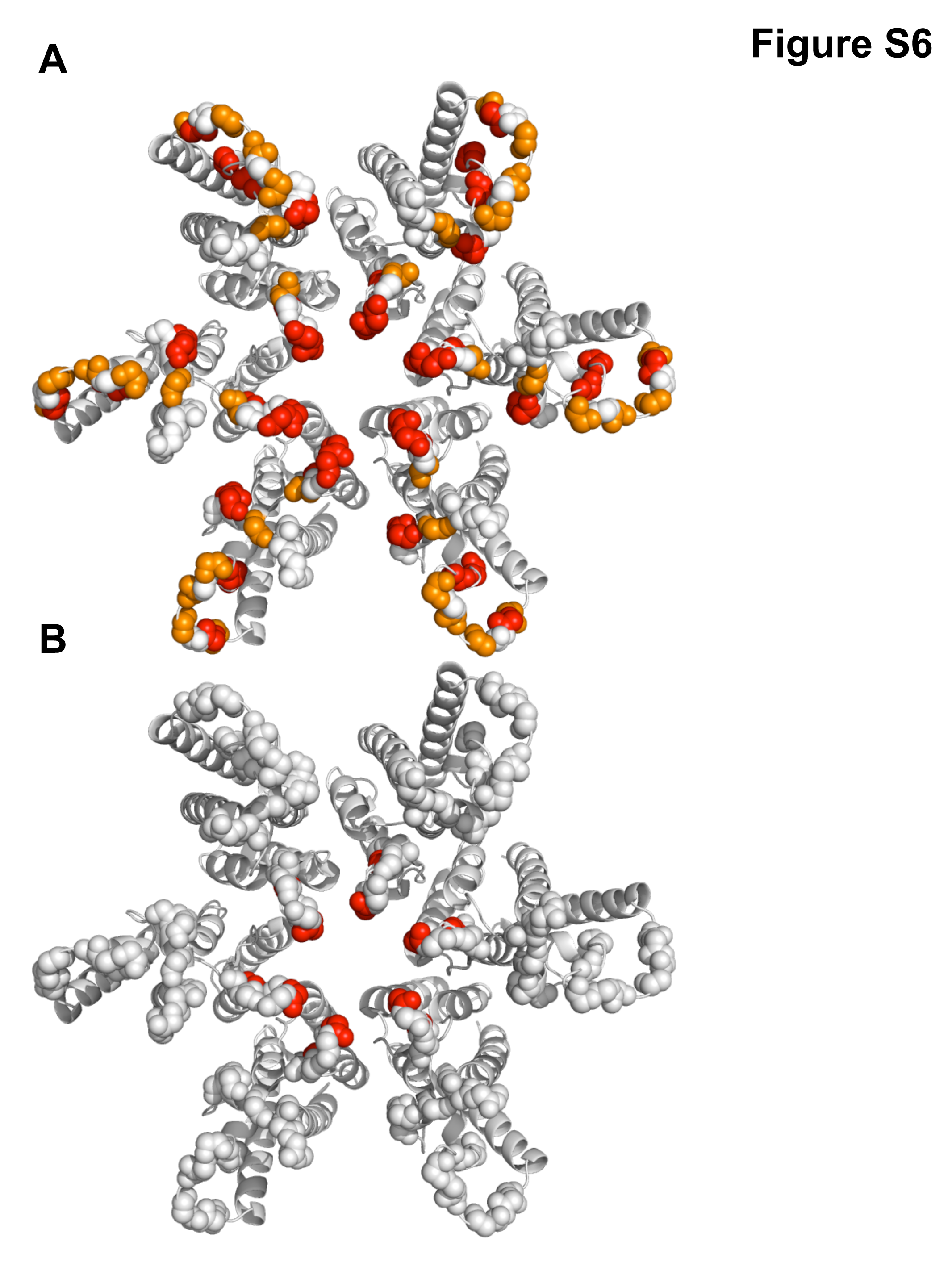

Supplement: Figure S6 — Mutations modulating Trim5α sensitivity mapped to the HIV-1 hexamer. Mutations from Table 1 mapped to the HIV-1 hexamer structure 3GV2. Restriction data for mutant viruses tested against the rhesus Trim5αTFP allele mamu1 (A) and the rhesus Trim5αQ allele mamu4 (B). Positions that were mutated on the capsid surface and were <2.5 fold more sensitive to Trim5α restriction than SIVmac239 are shown in gray spheres Orange spheres show the location of mutations associated with 2.5–5 fold gains in sensitivity to rhesus Trim5α. Red spheres indicate positions associated with >5 fold gains in sensitivity to rhesus Trim5α. Images created in PyMol (TIF) [file ppat.1003352.s006.tif]

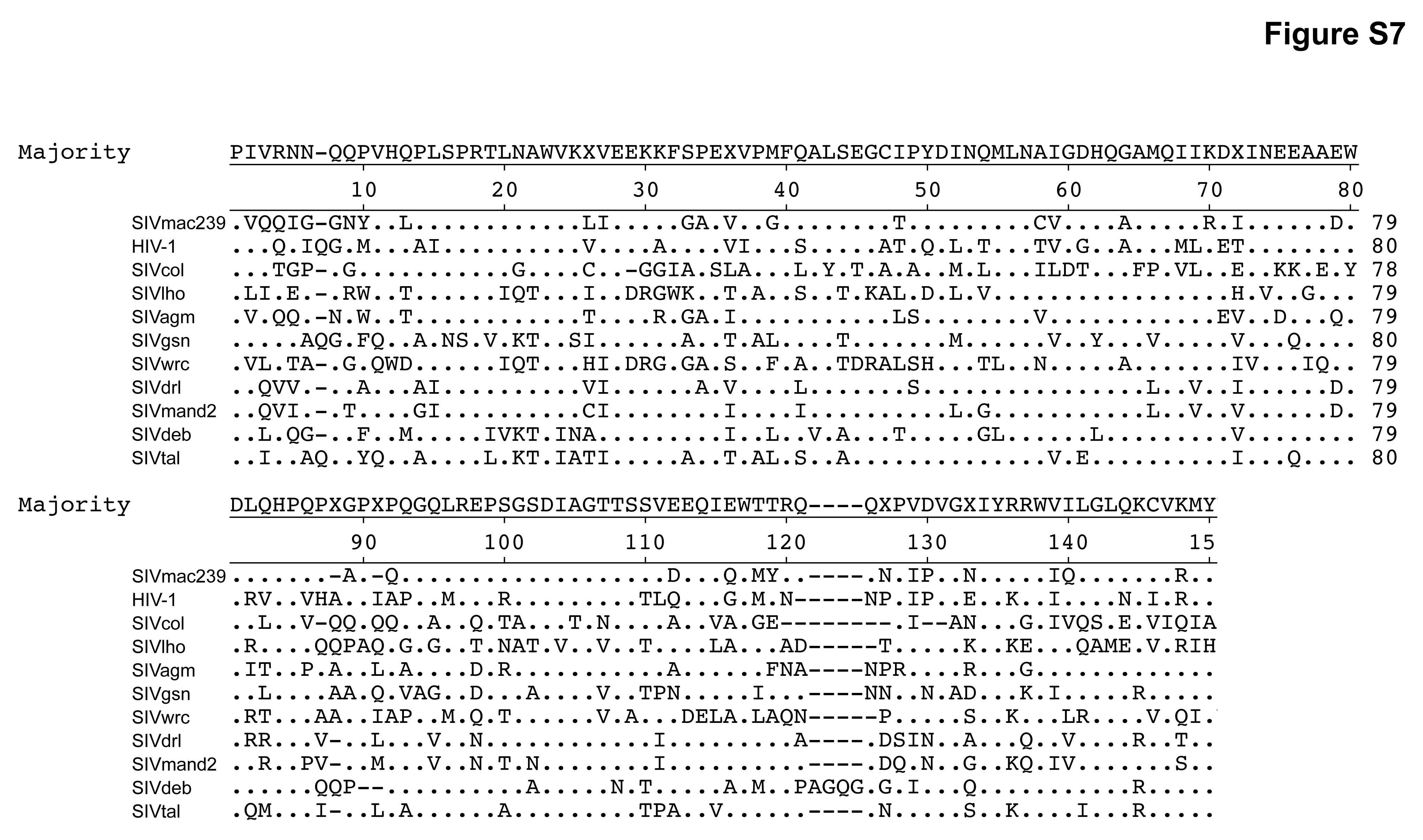

Supplement: Figure S7 — Amino acid alignment of divergent primate lentiviruses. Primate lentiviruses from eleven different lineages are aligned corresponding to the published alignment found in the Los Alamos Sequence database. Accession numbers: SIVmac239-M33262, HIV-1-K03455, SIVcol-AF301156, SIVlho-AF075269, SIVagm-U58991, SIVgsn-AF468658, SIVwrc-AM745105, SIVdrl-AY159321, SIVmand2-AY159322, SIVdeb-AY523865, SIVtal-AM182197 (TIF) [file ppat.1003352.s007.tif]

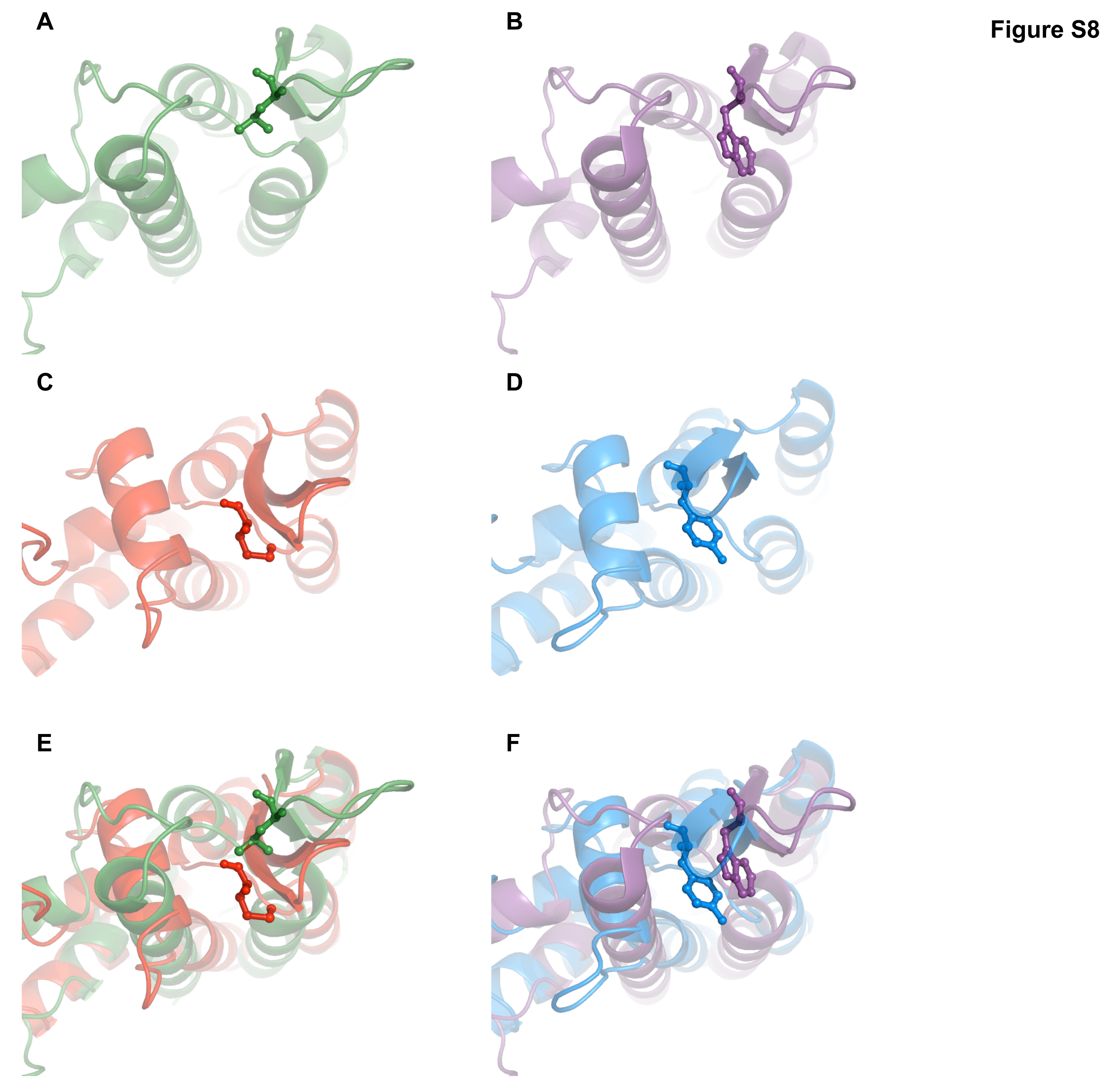

Supplement: Figure S8 — Structural comparison between SIVmac239 and MLVs with differential restriction by rhesus Trim5α. (A) β-hairpin of N-Tropic MLV (PDB: 1U7K) with residue L10 shown in sticks and spheres (B) β-hairpin of the N-MLV L10W mutant (PDB:2Y4Z) that is rhesus Trim5αTFP resistant, 10W shown in sticks and spheres. (C) β-hairpin of HIV-1 (PDB:2X2D) with M10 shown in sticks and spheres. (D) SIVmac239 β-hairpin Y9 shown in sticks and spheres. (E) Structural alignment of rhesus Trim5α sensitive N-MLV with HIV-1. (F) Structural alignment of the rhesus Trim5α resistant N-MLV L10W with SIVmac239. Images created in PyMol (TIF) [file ppat.1003352.s008.tif]

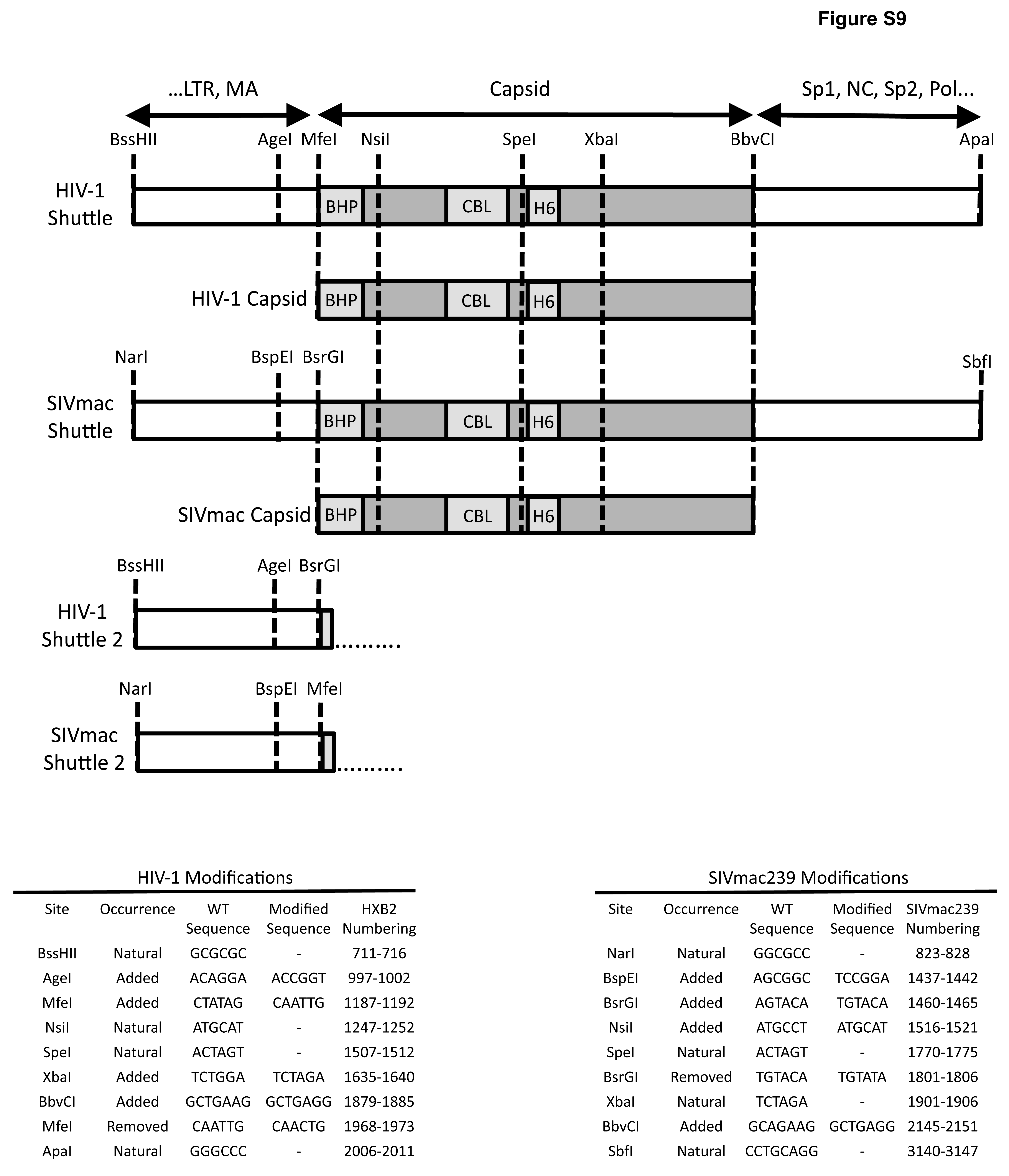

Supplement: Figure S9 — Schematic of synthesized genes and cloning strategy used to generate chimeric viruses. All constructs were synthesized by GENEART (Regensburg, Germany). Numbering corresponds to the standard HXB2 and SIVmac239 numbering, respectively. For efficient exchange of capsids between viruses and chimerization within capsids silent nucleotide changes were made in both viruses creating identical restriction sites. Naturally occurring restriction sites at the ends of the shuttle vector are used for insertion into the proper parental virus. Amino acid differences at the N-terminus of the CA protein did not allow us to use a single common enzyme for this site. Instead SIVmac239 constructs use a BsrGI site while HIV-1nl4.3 constructs use an MfeI site. Two additional shuttle vectors were made to accommodate either N-terminus in both SIVmac239 and HIV-1nl4.3 backbones. (TIF) [file ppat.1003352.s009.tif]
