# Supplementary material for: Gain-of-Sensitivity Mutations in a Trim5-Resistant Primary Isolate of Pathogenic SIV Identify Two Independent Conserved Determinants of Trim5α Specificity
Source: PLoS Pathog. 2013 May 9;9(5):e1003352. doi: 10.1371/journal.ppat.1003352 (PMC3649984; doi:10.1371/journal.ppat.1003352)
Supplement: Table S1 — Crystallography refinement statistics. (PDF) [file ppat.1003352.s010.pdf]

Table S1: Data collection and Refinement Statistics

	SIVmac239 CA NTD
Data Collection	*APS ID-24-E*
Resolution, Å	*50-2.9 (2.95-2.90)*
Wavelength (Å)	*0.9792*
Space Group	*P212121*
Unit cell dimensions (a, b, c), Å	*24.61, 56.66, 102.57*
Unit cell angles (α, β, γ) °	*90,90,90*
mean I/σ	*17.17 (3.0)*
Rsym	*0.063 (0.365)*
Completeness, %	*99.46 (95.10)*
Number of reflections	*15,453*
Redundancy	*4.4*
Refinement	
Resolution, Å	*2.9*
Number of reflections:	
Working	*3,345*
Free	*152*
Rwork, %	*25.4*
Rfree, %	*29.2*
Ramachandran plot,	
% favored	*89.1 (123/138)*
% disallowed	*0.72 (1/138)*
Rmsd bond lengths, Å	*0.0111*
Rmsd bond angles, °	*1.37*
Average B-factor	*109.3*

Note: values in parentheses denotes highest resolution shell
